# Sociocultural and ecological factors influencing management of edible and non-edible plants: the case of Ixcatlán, Mexico

**DOI:** 10.1186/s13002-017-0185-4

**Published:** 2017-10-30

**Authors:** Selene Rangel-Landa, Alejandro Casas, Eduardo García-Frapolli, Rafael Lira

**Affiliations:** 1 Instituto de Investigaciones en Ecosistemas y Sustentabilidad, UNAM, Antigua Carretera a Pátzcuaro 8711, 58190 Morelia, Michoacán Mexico; 2 UBIPRO, Facultad de Estudios Superiores Iztacala, UNAM, Av. de los Barrios #1, Los Reyes Ixtacala, Mexico, Mexico

**Keywords:** Cultural importance, Domestication, Ixcatec, Plant management, Risk management, Reciprocity interchange, Spiritual values and plant management, Tehuacán Valley

## Abstract

**Background:**

Identifying factors influencing plant management allows understanding how processes of domestication operate. Uncertain availability of resources is a main motivation for managing edible plants, but little is known about management motives of non-edible resources like medicinal and ceremonial plants. We hypothesized that uncertain availability of resources would be a general factor motivating their management, but other motives could operate simultaneously. Uncertainty and risk might be less important motives in medicinal than in edible plants, while for ceremonial plants, symbolic and spiritual values would be more relevant.

**Methods:**

We inventoried edible, medicinal, and ceremonial plants in Ixcatlán, Oaxaca, Mexico, and conducted in-depth studies with 20 native and naturalized species per use type; we documented their cultural importance and abundance by interviewing 25 households and sampling vegetation in 33 sites. Consumption amounts and preferences were studied through surveys and free listings with 38 interviewees. Management intensity and risk indexes were calculated through PCA and their relation analyzed through regression analyses. Canonical methods allowed identifying the main sociocultural and ecological factors influencing management of plants per use type.

**Results:**

Nearly 64, 63, and 55% of all ceremonial, edible, and medicinal wild plants recorded, respectively, are managed in order to maintain or increase their availability, embellishing environments, and because of ethical reasons and curiosity. Management intensity was higher in edible plants under human selection and associated with risk. Management of ceremonial and medicinal plants was not associated with indexes of risk or uncertainty in their availability. Other sociocultural and ecological factors influence management intensity, the most important being reciprocal relations and abundance perception.

**Conclusions:**

Plant management through practices and collectively regulated strategies is strongly related to control of risk and uncertainty in edible plants, compared with medicinal and ceremonial plants, in which reciprocal interchanges, curiosity, and spiritual values are more important factors. Understanding how needs, worries, social relations, and ethical values influence management decisions is important to understand processes of constructing management strategies and how domestication could be started in the past and are operated at the present.

## Background

Management of plant resources and traditional ecological knowledge (TEK) are intimately related biocultural aspects that crucially influence the modeling of strategies of multiple use of natural resources in rural communities [1–3]. Understanding how management systems do operate, and identifying the factors influencing and motivating them, is greatly important for analyzing how and why plant management is currently decided, how the ongoing processes of domestication are operating, and how these could have operated in the past [4]. Therefore, studies of these processes may be relevant for designing current strategies of sustainable use of plant resources and ecosystems, as well as for understanding factors that led humans to start domestication and agriculture in the past.

Management can be defined as all practices, interventions, transformations, strategies, or decisions deliberately made by humans on ecosystems, their components, functions, and even their emergent properties, in order to use, conserve, or recover them [5, 6]. In traditional contexts, management practices are based on ancient knowledge transmitted from generation to generation, but innovations are continually constructed influenced by new observations, experimentation, and information from recent sources (information from neighboring villages, schools, communication media, interventions by NGOs, governmental promoters, researchers, among others). Both old and new management practices are organized in dynamic systems of knowledge, beliefs, cultural and spiritual values, and local institutions [7, 8].

For studying domestication, it is particularly interesting to document the morphological and genetic divergences between wild and managed populations directed to maintain or increase the availability of particular phenotypes of managed species. Such aspects provide valuable elements for explaining how processes of domestication currently operate and how these could have operated in the past. The ongoing processes of domestication can be documented in numerous rural communities of the world and are responsible for a continuous mechanism of divergence and generation of a new variation of genetic resources. As a research group, we have focused our attention on domestication processes occurring in Mesoamerica, one of the most active areas of plant management and one of the earliest centers of plant domestication in the World [5, 9, 10]. Numerous studies have documented the consequences of domestication, but relatively few have analyzed what factors motivate people to manage and domesticate plants, animals, and other organisms. In this study, we focus our attention to analyzing the main causes of the process.

Management involves several types of practices, tools, and relations between energy invested and amounts of resources obtained; such aspects reflect different degrees of management intensity [11, 12]. Authors analyzing this topic coincide that management intensity of plants goes from gathering, let standing, special care, protection, and transplanting, to practices procuring increase of desirable plant abundance by enhancing and deliberately propagating them [5, 6]. Some variables have been proposed as relevant for analyzing the degree of management intensity: (1) the number and complexity of practices carried out, (2) the number of people or social units (i.e., persons, households, or communities) participating in such practices, (3) the involvement and level of complexity of planning strategies, (4) social agreements regulating the actions, (5) the occurrence of human selection favoring particular phenotypes and the intensity in which it operates, (6) the deliberate practices favoring human-mediated gene flow and manipulation of plant reproduction, (7) the amounts of fossil or human energy invested in practices, (8) the complexity of tools used, and (9) the amount of products obtained per area unit [11–13].

In several case studies with cacti, agaves, herbs, and trees, mainly with edible use, we have documented that managed plants under higher management intensity are those more consumed or commercialized and whose future availability becomes compromised due to their relatively low availability in relation to the demand on them [11–17]. In other words, plant management is influenced by the amounts of products required by social units (which is in turn influenced by their cultural and economic value) but also by people’s perception of the product quality and their substitutability or not by other resources. In addition, management is influenced by the natural availability of plant products, determined by parameters like distribution and abundance, their resilience capacity after human impact on populations, their vulnerability, and management feasibility [11, 12, 18], as well as the ease of access to resources regulated by land tenure and communitarian agreements. All these relations have allowed proposing that management is a response to the need of facing risks or uncertainty in the current and future availability of resources [12]. In other words, it is a response of people’s worries for ensuring availability of resources [12, 19] or preventing their loss [15].

However, some studies have documented that cultural motives such as relations of reciprocity among persons and communities, some spiritual aspects, and efforts to maintain customs and traditions [20, 21] commonly motivate management practices. In addition, practices such as tolerance or let standing of plants in disturbed areas may be associated with ethical principles like the right of plants to live, whereas enhancing abundance of some species may be associated to favor variants of higher quality to embellish the sites where they occur [22–26]. Transplanting and other forms of propagation may simply be motivated by the need to have particular plants closer because of their beauty, odor, and role in rituals or simply because of curiosity to know how plants grow and reproduce [19, 23]. These scenarios allow supposing that management type and intensity are not only responses to risk, but also practices related to ethic or esthetic values, symbolism, or curiosity, and all factors may be operating simultaneously. Analyzing how people make management decisions on plants with different purposes may allow visualizing more clearly different motives for managing plants and management intensity [12, 27]. Therefore, this study explores management motives for plants with different use types.

We hypothesized that uncertainty in availability of plant resources is a main factor motivating management of plants, especially those directed to satisfy basic needs. We therefore expected that edible plants would have higher management intensity as the higher the risk or uncertainty in their availability, as similarly documented in previous studies [12, 13]. Uncertainty would be influenced by the scarcity of plant resources and human pressures on them; therefore, scarce species with high cultural value would be more intensely managed. Ecological aspects of plants like survival, vigor, or resprouting capacities, which may be affected by use, and others that influence the ease of management like life cycle length, reproductive systems, ease of propagation, and adaptability to human-made environments would influence management types and intensities. Medicinal plants are generally used in smaller amounts than edible plants (except those that are extracted for commercialization); therefore, we expected that the pattern of management as a response to risk would be less pronounced than in edible plants [12]. Finally, we expected that the management of plants used for rituals and ceremonies, is not necessarily influenced by risk since purposes and amounts of plants used for these purposes follow different rationalities in which reciprocity relations, esthetic and symbolic values could be important.

Summarizing, our study aimed to analyze how management type and intensity are influenced by sociocultural and ecological factors in edible, medicinal, and ceremonial plants among the Ixcatec from Santa María Ixcatlán, Mexico. We analyzed whether or not people’s worries about availability of plants operate similarly in plants with different use type and look for evaluating the weight of different motives for decisions on managing plant resources.

## Methods

### Study area

**Fig. 1:**
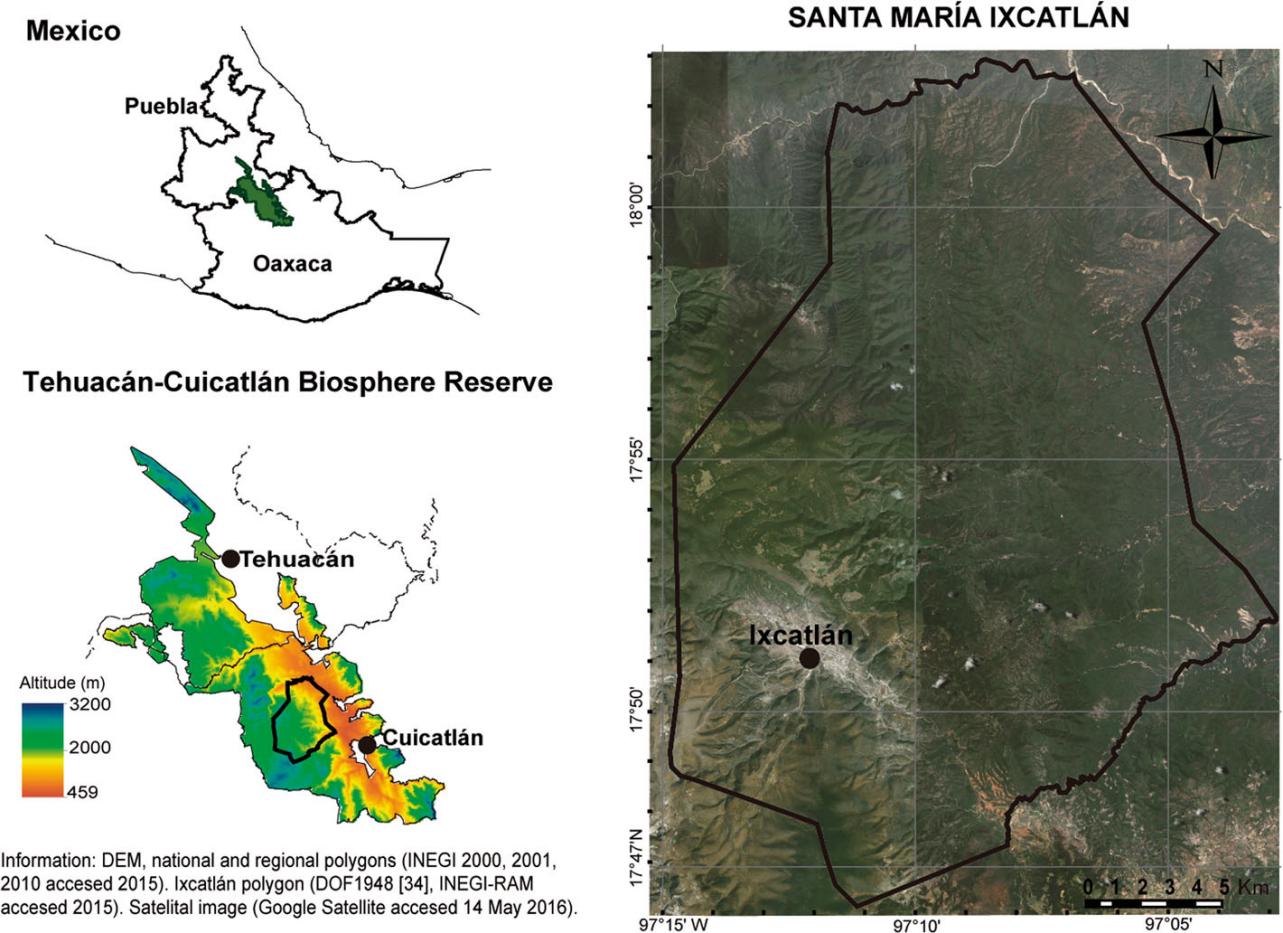
Location of the community of Santa María Ixcatlán, Oaxaca, Central México

Santa María Ixcatlán belongs to the Tehuacán-Cuicatlán Biosphere Reserve, Central México (Fig. 1). It is located at elevations from 800 to 2600 m, with annual rainfall of 721 mm and average temperature of 17.2 °C. Climate is temperate sub-humid in high zones and semiarid in lowlands [28, 29]. The traditional General Assembly regulates decisions on land, natural resources, and social life [30]. Ixcatlán is inhabited by 171 households [31], almost all of them catholic [26]; 80% of the people consider themselves to be indigenous, but only 15 persons speak Ixcatec, and this is the only village of the world where the Ixcatec language is spoken [31, 32]. Subsistence of the people is based on the multiple use of natural resources and ecosystems, seasonal agriculture, livestock raising, and forest resource extraction [26]. We previously reported 630 plant species used by local people for satisfying different needs [26], nearly 400 species receiving some type of management in order to increase their abundance [26]. Gathering and management of plants is carried out in 18 types of forests, agroforestry systems, and homegardens over a 41,530-ha territory [26, 31–35].

### Inventory of edible, medicinal, and ceremonial plants

Ethnobotanical studies by Rangel-Landa et al. [26] documented names, uses, and management of all plant species through semi-structured interviews with 44 persons (see Table 1) in 73 sessions. The information was systematized into the ethnobotanical database of Mexican plants (BADEPLAM), at the Botanical Garden, UNAM, and voucher specimens were deposited in the herbaria MEXU, EBUM, IEB-Bajío, and IBUG. The nomenclature of plant species followed APG III consulted through the site www.theplantlist.org [36].

**Table 1:** Consultants’ details and the activities in which they collaborated

ID	Sex	Age	Language	Main activities	Participant type	Semiestructured interviews	Free lists	Surveys 2012	In-depth interviews	Homegarden	Agricultural field	Mescal factory
1	Male	25	Spanish	Mescal production	Key participant	Yes	Yes					2
2	Male	50	Spanish	Agriculture, mescal production	Key participant	Yes		16			1	
3	Male	72	Spanish, Ixcatec	Agriculture, palm weaver							6	
4	Male	66	Spanish	Agriculture, palm weaver				12				
5	Male	46	Spanish	Agriculture, palm weaver	Key participant	Yes	Yes				2	
6	Female	44	Spanish	Domestic chores, palm weaver		Yes		15		8		
9	Male		Spanish	Agriculture, palm weaver		Yes				5		
10	Male	48	Spanish	Agriculture, commerce	Key participant	Yes	Yes	1				
11	Male	62	Spanish	Agriculture, mescal production	Key participant	Yes				10		
12	Male	35	Spanish	Agriculture, mescal production, palm weaver				13				
14	Male	67	Spanish	Agriculture, palm weaver							3	
16	Male	73	Spanish	Agriculture, mescal production, palm weaver			Yes					
17	Female		Spanish	Student			Yes					
18	Female	60	Spanish	Domestic chores, palm weaver		Yes				13		
19	Female	35	Spanish	Domestic chores, palm weaver				1				
20	Female	62	Spanish	Domestic chores, palm weaver	Key participant	Yes		7	Yes	15		
23	Male	72	Spanish	Agriculture, palm weaver			Yes			3		
24	Male	70	Spanish	Agriculture, palm weaver				14				
25	Male	51	Spanish	Agriculture, mescal production, palm weaver				5				
26	Male	82	Spanish, Ixcatec	Agriculture, palm weaver	Key participant	Yes					4	
27	Male		Spanish	Agriculture, palm weaver				6				
28	Male	68	Spanish	Agriculture, palm weaver				4				
30	Male	59	Spanish	Agriculture, mescal production, palm weaver			Yes					
31	Male	57	Spanish	Shepherd	Key participant	Yes	Yes		Yes			
34	Female	48	Spanish	Domestic chores, palm weaver				5				
35	Male		Spanish	Student			Yes					
36	Female	70	Spanish	Domestic chores, palm weaver		Yes				7		
37	Female	46	Spanish	Commerce, domestic chores			Yes		Yes			
38	Female	18	Spanish	Student		Yes						
39	Female		Spanish	Domestic chores, palm weaver			Yes	20				
41	Female	34	Spanish	Domestic chores, palm weaver			Yes		Yes			
42	Female	64	Spanish, Ixcatec	Domestic chores, palm weaver	Key participant	Yes	Yes	12		17		
43	Male		Spanish	Agriculture, mescal production, palm weaver		Yes						
46	Male	54	Spanish	Agriculture, palm weaver				15				
47	Female	41	Spanish	Domestic chores, palm weaver					Yes	10		
48	Female		Spanish	Domestic chores, palm weaver		Yes				12		
49	Male		Spanish	Commerce		Yes						
50	Female	41	Spanish	Commerce, domestic chores			Yes					
51	Female	35	Spanish	Domestic chores, palm weaver				6				
52	Male	76	Spanish, Ixcatec	Agriculture, palm weaver	Key participant	Yes	Yes	9		16		
53	Male		Spanish	Agriculture, palm weaver						12		
55	Male	32	Spanish	Agriculture, construction worker, palm weaver			Yes					
57	Female		Spanish	Domestic chores, palm weaver		Yes				9		
58	Female	41	Spanish	Domestic chores, palm weaver	Key participant	Yes	Yes	16				
59	Male	38	Spanish	Agriculture, palm weaver			Yes					
60	Female	83	Spanish	Domestic chores, palm weaver				11				
61	Female	88	Spanish, Ixcatec	Domestic chores, palm weaver				2				
63	Female	59	Spanish	Domestic chores, palm weaver		Yes					7	
64	Male		Spanish	Agriculture, mescal production, palm weaver, shepherd		Yes						
65	Male	73	Spanish	Agriculture, palm weaver		Yes		18	Yes			
66	Female	51	Spanish, Ixcatec	Domestic chores, palm weaver	Key participant	Yes	Yes	2				
67	Male	20	Spanish	Agriculture, palm weaver			Yes					
68	Male	61	Spanish	Agriculture, palm weaver		Yes						
69	Male		Spanish	Student			Yes					
70	Female	71	Spanish, Ixcatec	Domestic chores, palm weaver	Key participant	Yes	Yes	18		4		
72	Male	86	Spanish	Agriculture, palm weaver				11				
73	Female	82	Spanish, Ixcatec	Domestic chores, palm weaver	Key participant	Yes				5		
74	Male	38	Spanish	Agriculture, construction worker, palm weaver			Yes					
76	Female	65	Spanish	Domestic chores, palm weaver		Yes	Yes			3		
77	Male	38	Spanish	Agriculture, palm weaver			Yes					
78	Female	40	Spanish	Domestic chores, palm weaver				10				
79	Male	59	Spanish	Agriculture, mescal production, palm weaver, construction worker			Yes	20	Yes			5
81	Female		Spanish	domestic chores, palm weaver			Yes					
82	Female	62	Spanish	Domestic chores, palm weaver		Yes				2		
83	Female	33	Spanish, Ixcatec	Domestic chores, palm weaver			Yes		Yes			
84	Male	14	Spanish	Palm weaver, student	Key participant	Yes				6		
85	Male		Spanish	Mescal production, palm weaver		Yes						
87	Female		Spanish	Domestic chores, palm weaver		Yes				9		
88	Male	57	Spanish	Agriculture, palm weaver			Yes					
89	Female	72	Spanish	Domestic chores, palm weaver			Yes					
90	Male	26	Spanish	Agriculture, palm weaver			Yes		Yes			
91	Male	80	Spanish	Agriculture, palm weaver			Yes	19				
93	Female	66	Spanish, Ixcatec	Domestic chores, palm weaver, shepherdess	Key participant	Yes	Yes	17		1		
95	Male	64	Spanish	Agriculture, mescal production, palm weaver	Key participant	Yes	Yes	7				
97	Female	79	Spanish, Ixcatec	Domestic chores, palm weaver	Key participant	Yes		14		11		
98	Male	88	Spanish, Ixcatec	Palm weaver	Key participant	Yes		3		14		
100	Female	84	Spanish, Ixcatec	Domestic chores, palm weaver	Key participant	Yes	Yes	4				
101	Female	94	Spanish, Ixcatec	Domestic chores, palm weaver		Yes						
102	Female	33	Spanish	Domestic chores, palm weaver				13				
103	Male	25	Spanish	Agriculture, shepherd	Key participant	Yes	Yes			15		
104	Female	39	Spanish	Domestic chores, palm weaver		Yes				1		
106	Male	55	Spanish	Agriculture, mescal production, palm weaver			Yes					
107	Male		Spanish	Agriculture				8				
108	Female	92	Spanish, Ixcatec	Domestic chores, palm weaver		Yes						
109	Female	32	Spanish	Domestic chores, palm weaver			Yes		Yes			
110	Female		Spanish	Nurse		Yes						
111	Female	24	Spanish	Domestic chores, nurse assistant		Yes						
113	Female	48	Spanish	Domestic chores, palm weaver		Yes			Yes	6		
114	Female	50	Spanish	Domestic chores, palm weaver					Yes			
115	Male	57	Spanish	Agriculture, palm weaver					Yes			
116	Female	55	Spanish	Domestic chores, palm weaver					Yes	18		
117	Male	37	Spanish	Agriculture, palm weaver					Yes			
118	Male	31	Spanish	Agriculture, palm weaver					Yes			
119	Female	46	Spanish	Domestic chores, palm weaver					Yes			
120	Female	35	Spanish	Domestic chores, palm weaver					Yes			
121	Male	39	Spanish	Agriculture, construction worker, palm weaver					Yes			
122	Female	71	Spanish	Domestic chores, palm weaver					Yes			
123	Female	74	Spanish	Domestic chores, palm weaver					Yes	20		
124	Female		Spanish	Domestic chores, palm weaver					Yes			
125	Female	81	Spanish	Domestic chores, palm weaver					Yes	21		
126	Female	70	Spanish	Domestic chores, palm weaver					Yes			
127	Female	31	Spanish	Domestic chores, commerce					Yes			
129	Male		Spanish	Agriculture, mescal production								3
130	Male		Spanish	Agriculture, mescal production								4
131	Male		Spanish	Agriculture, mescal production								1

### In-depth interviews and surveys

In order to analyze how management is influenced by sociocultural and ecological factors, we selected samples of edible, medicinal, and ceremonial plants. The samples included 20 species of native and naturalized plants per use type, representing the management intensity gradient [26].

In-depth interviews were conducted to obtain deeper and detailed information on uses, values, perception about availability, vulnerability, and management practices (Table 2) for the selected species. These interviews were conducted with 25 persons selected at random (17 women and 8 men, see Table 1). In order to estimate the proportion of families that consume the studied plants in the village, we conducted a survey documenting the role of plant resources in people subsistence [26]. The survey included 20 households selected at random.

**Table 2:** Criteria of variables considered for analyzing sociocultural and ecologic factors that influence management intensity

Matrix	Variables	Description	Criterion and values
Sociocultural (matrix X)	Uses number	Total number of registered uses	1 per use
	SI basic plants	Sutrop’s cognitive prominence index of plants considered as basic to live in Ixcatlán	0–1; 0 is a value assigned when no consultant mentioned the plant, and 1 is a theoretical value that a plant could have if all consultants mentioned it at first rank [39]
	SI by use type	Sutrop’s cognitive prominence index of plants by category (edible, medicinal, ceremonial)	0–1; 0 is a value assigned when no consultant mentioned the plant, and 1 is a theoretical value that a plant could have if all consultants mentioned it at first rank [39]
	Consumption	Proportion of families that have consumed the species for the analyzed use in the last 2 years	0–1
	Use frequency	Frequency of consumption per availability season/year for analyzed use (2)	0 = never been consumed; 1 ≤ 5 times in their life; 2 ≥ 5 times in their life but not regularly; 3 = 1 time every 2 availability seasons; 4 = 1 a 2 times by availability season; 5 = 3 a 10 times by availability season
	Recognized variants	Types or varieties recognized (1)	0 = no varieties are recognized; 1 = varieties are recognized for a plant, but each variety is a different species; 2 = varieties are recognized for a species but are used equally; 3 = varieties are recognized and have specialized use
	Economic interchange	Type of commercial exchange (1)	1 = direct consumption; 2 = bartering; 3 = sold inside the village by collectors of the community or comers who obtain it in other places; 4 = harvested inside the village and are marketed outside (plants or products)
	Reciprocity interchange	Type of exchange of reciprocity (1)	1 = direct consumption; 2 = it is given and received as a gift to/from others; 3 = it is offered in communal celebrations (harvested by sponsors celebration or families who offer the plants to sponsor celebration)
	Sociocultural strategies	Strategies to obtain the plant when scarce or unavailable (1)	0 = nothing; 1 = mobility, look elsewhere; 2 = substitution for other species or products; 3 = store them; 4 = ask someone to give them; 5 = seek to obtain it by barter; 6 = buy them
	Useful parts^a^	Number of useful parts	1 per used part
	Harvest effort^a^	Invested effort in harvest in a journey (1)	1 = opportunist; 2 = journey dedicated to harvest the species
	Tools for harvest^a^	Use of tools, supplies, and vehicles in harvest (1)	None, only hands are used; 1 = objects obtained at harvest site; 2 = knife, machete; 2 = *Arundo donax* pole, baskets, bags; 3 = load animals, vehicles, chainsaws
Ecological (Matrix W)	Abundance perception	Abundance perception in the territory (2)	1 = very abundant; 2 = abundant; 3 = regular abundance; 4 = scarce; 5 = rare
	Vulnerability	Plant vulnerability to factors affecting productivity, quality, and survival (2)	1 = nothing affects and always produces the same; 2 = plague, drought, steady harvest, others
	Life cycle	Life cycle type of the species	1 = annual; 2 = perennial
	Reproduction	Reproduction type of the species	1 = sexual and asexual; 2 = sexual
	Harvested parts	Harvested parts for all use types of the plant in function of survival, resprouting, and reproductive capacity after useful part harvest (1)	1 = living individual; 1 = dry branches; 2 = exudates, thorns; 3 = leaves; 4 = sprout; 5 = mature branches (lignified tissue/flowers); 6 = fruits, seeds; 7 = bark; 8 = all flowers/fruits of the season; 9 = main stalk; 9 = roots; 10 = complete individuals
	Nearness to harvest site^a^	Closeness perception of harvest sites to consumption site (2)	1 = far away; 2 = far; 3 = not too far; 4 = near; 5 = at hand
	Temporal availability^a^	Temporal availability of the useful part for the analyzed use (2)	1 = all year; 2 = months; 3 = weeks; 5 = days
Management (Response matrix Y)	Collective regulations	Type of regulation for the harvest (1)	0 = without restrictions; 1 = there are “costumbres” traditions that indicate the techniques, quantity, and occasions of harvest; 2 = in addition to communal agreements aimed at regulating the access, they are aware that external institutions protect the species; 3 = complaints have been made or penalties imposed
	Management practices	Management practice type (1)	1 = gathering, forage; 2 = gathering with care to avoid damaging the plant; 3 = tolerance; 4 = enhancement; 5 = protection; 6 = transplanting of individuals; 7 = propagation
	Artificial selection	Selection of individuals and propagules (1)	0 = without selection; 1 = selection of individuals or parts that are collected for consumption; 2 = selection of tolerated, protected or enhanced individuals; 3 = selection of individuals from which seeds or cuttings are obtained to propagate
	Management in AFS	Species presence proportion in homegardens, agricultural fields, and mescal factories	0–3
	Practices number^a^	Number of management practices carried out	1 by type of practices
	Maintaining labors^a^	Type of labors carried out to protect, enhance, and cultivate	1 = prepare soil; fix to hosts; exclusion of predators with fences, cages; removal of competitors; pruning, removing dried or diseased leaves; mechanical support; addition of forest soil, sand, ash, residues of organic matter; addition of lime 2 = irrigation 3 = infrastructure and special equipment for maintenance
	Management system type^a^	System type where plant is managed with respect to species natural distribution (1)	1 = wild vegetation where plants are distributed naturally; 2 = homegardens, gardens in mescal factories or “palenques,” and agricultural fields, where plants are naturally distributed; 3 = homegardens, mescal factories, and agricultural fields, where plants have been carried; 4 = greenhouses and nurseries

Numbers in variable description indicate the following: (1) addition of the different values registered for the species; (2) average of category values mentioned by consultants

^a^Variables not included in principal component analysis and partial canonical analysis

### Free listing

For exploring the use preferences of the plants studied, we included different valuing criteria (utilitarian, symbolic, esthetic, and emotional) through the free listing technique [37, 38]. We interviewed 38 persons (22 men, 16 women, Table 1) [26], asking them to list plants used: (1) in ceremonies and offerings to Saints and dead people, (2) as food, (3) for health care [26], and (4) for satisfying basic needs, those considered indispensable to live. We estimated their cognitive prominence for each use type through the formula *S* = *F*/(*N* mP), where *F* is the frequency of each plant species, *N* the number of people interviewed, and mP the average position in which a plant was named [39]. The index was calculated with Flame v1.0 [40].

### Vegetation sampling

In order to identify the places where the plant species studied are managed, and how abundant they are in forests and agroforestry systems (AFS), we sampled vegetation in 7 agricultural plots, 21 homegardens, and 5 AFS associated to sites of mescal production [26].

### Selection of variables for the analyses

Socio-ecological and technological variables were selected based on our previous studies [11, 12], which were organized in three main data matrixes. One matrix was with information on indicators of social, cultural, and economic importance of the species studied. A second matrix had information on biological aspects (life cycle length, types of reproduction, growth patterns, among others) and on people’s perception about the availability and vulnerability of each species. The third matrix had information about management practices and management intensity. Information on qualitative variables were categorized assigning numeric values from lower to higher management intensity according to the complexity of strategies and practices, occurrence or not of human selection, and low to high number of persons involved in the management type, among others. We also categorized from lower to higher social, cultural, and economic importance, considering that the higher their importance, the higher the potential risk associated to human pressure. Finally, we categorized from lower to higher vulnerability associated with biological characters considering the impact of human extraction of resources on individual plants and populations (Table 2). We averaged values of different categories, and in variables involving counting or binary records, we calculated the proportions of the states (Table 2). We excluded highly correlated variables, selecting those better representing the importance and management intensity of the plant species analyzed (Table 2).

### Data analyses

In order to characterize the use and management of plants with edible, medicinal, and ceremonial uses, we used our previous data about all the species recorded [26] and the in-depth interviews for the selected species. We analyzed these data by cross-checking information and using descriptive statistics. We conducted principal component analysis (PCA) with data about management of all the native and naturalized species in order to classify management intensity among use types. The scores of the first principal component were used as management intensity index [12, 15]. We performed Kruskal-Wallis tests in order to identify differences among scores of management intensity of plants with ceremonial, edible, and medicinal uses. With the data of selected species, we performed two PCA per use type, one with the variables of the management type matrix and the other with the sociocultural and ecological variables (Table 2); the scores were used as an index of management intensity and a risk index, respectively. The relationships between risk and management intensity were analyzed through regression analyses.

Partial canonical analyses were performed using canonical correspondence analysis (CCA) per use type, in order to identify which fraction of the variation in plant management is explained by sociocultural and ecological factors and the effect of the interaction between the two types of variables [12, 15, 16, 41]. For each analysis, we used three matrices, a Y matrix containing the response management variables, an X matrix with sociocultural explanatory variables, and a W matrix of the ecological explanatory variables. Through this method, we conducted partial analyses with different combinations of the matrixes of the explanatory variables: (1) CCA for matrix Y, (2) CCA with matrix Y explained by matrix X, (3) CCA with matrix Y explained by matrix W, and (4) CCA with matrix Y explained by the combined effect of the W + X matrices. The total constrained eigenvalue of each analysis was tallied to evaluate how much the management intensity matrix is explained by the sociocultural and ecological variables.

For each analysis, the sum of all canonical eigenvalues divided by the sum of all canonical eigenvalues of the CA with management data allowed calculating the corresponding fraction of variation explained by the analysis. The significance of the models was estimated by permutation tests. All analyses were conducted through the R software [42]. In the PCA and CCA analyses of medicinal plants, *Agave potatorum* and *Quercus acutifolia* were excluded since these species were outliers.

## Results

### Ceremonial plants

**Fig. 2:**
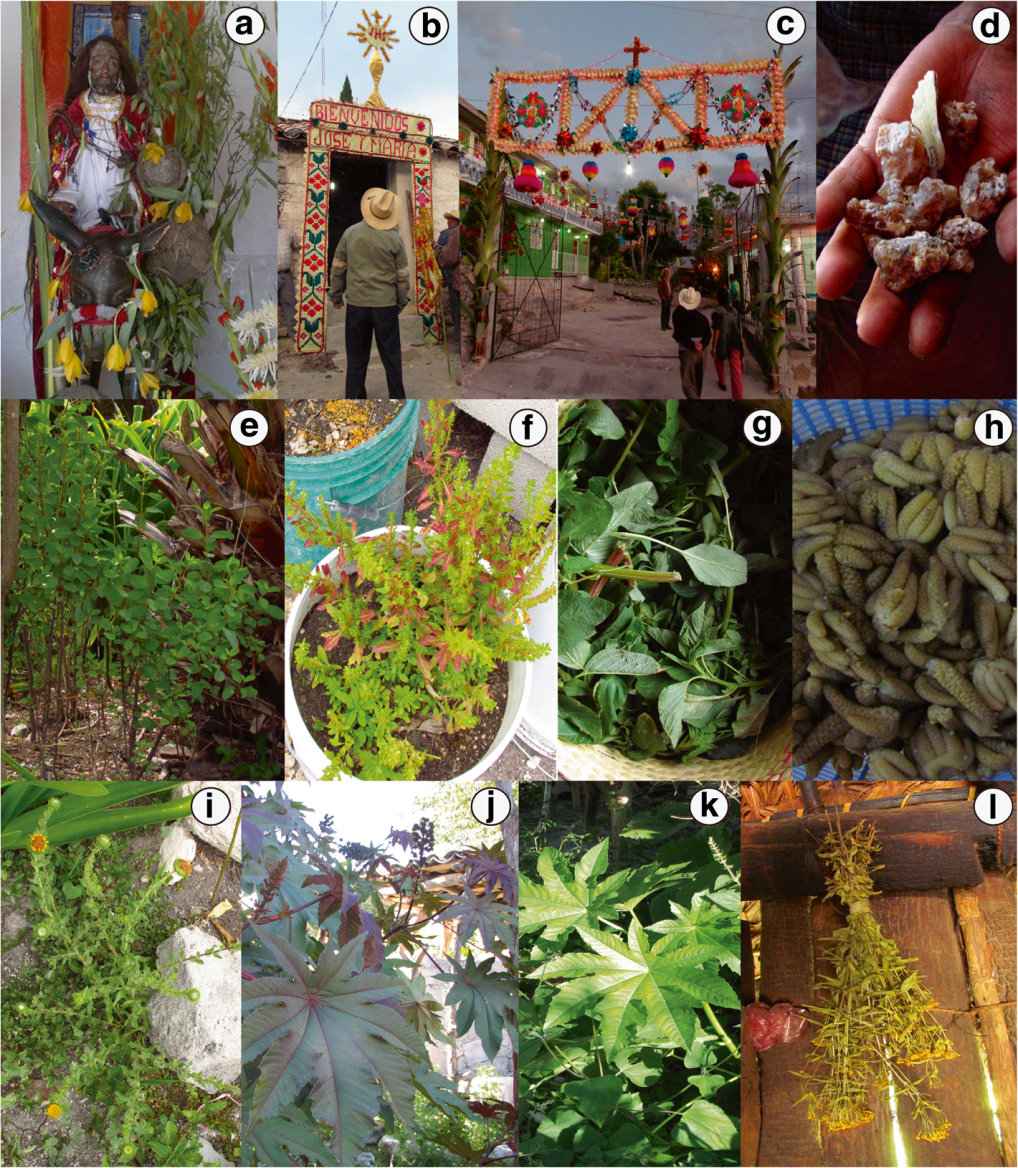
Ceremonial, edible, and medicinal plants of Santa María Ixcatlán community. **a** Offering “adornment” of *Brahea dulcis* leaves, *Euchile karwinskii* flowers, *Litsea glaucescens* branches, and wasp honeycombs to San Ramón in Palm Sunday celebration. **b** *Beaucarnea stricta* arch to welcome the Saints in “posadas” celebrations. **c** *Tillandsia grandis* and *Dasylirion serratifolium* arch to welcome the Saints in “posadas” celebrations. **d** *Bursera biflora* resin. **e** *Porophyllum ruderale subsp*. *macrocephalum* cultivated in a homegarden. **f** *Dysphania ambrosioides* transplanted in a bucket to protect it from animals and to facilitate its care. **g** Tender branches of *Amaranthus hybridus* collected during agricultural labor.; **h** Boiled floral buds of *Dasylirion serratifolium*. **i** *Grindelia inuloides* plant cultivated in a homegarden. **j** Red *Ricinus communis* variety managed in a homegarden. **k** White *Ricinus communis* variety. **l** Bunch of *Tagetes lucida* dry plants

We recorded 128 ceremonial plant species, 78 of them native or naturalized (Table 3); 22 species are considered by people to be basic for their life (Table 4). We recorded 48 species used for altars at homes for venerating Saints (Fig. 2). The most valuable species are those appreciated for their beauty and odor of their flowers (Table 5). As part of the communitarian celebrations, local people use 33 species as incense-like resin called copal (*Bursera* spp.), in the religious processions (*Litsea glaucescens*), and as ornaments offered to Saints (orchids, *Dasylirion serratifolium*, *Tillandsia grandis*, *Beaucarnea stricta*) (Fig. 2). *Brahea* spp. leaves blessed are used for weaving shoes for dead people. The copal resin is used in praying, altars, processions, masses, and funerary rites and for protecting against “aires” (negative feelings, dangerous situations that may cause illnesses or accidents).

**Table 3:** Management of native and naturalized species of Santa María Ixcatlán by use type

	Ceremonial	Edible	Medicinal
Only gathered	28	30	81
Tolerated	21	40	79
Enhancement	6	7	9
Protection	37	28	31
Transplanting	26	20	15
Propagation	18	11	12
Total	77	80	178

**Table 4:** Native and naturalized plants of Santa María Ixcatlán with ceremonial, edible, and medicinal use

Family	Species	Voucher number^a^	Common name	Ceremonial use	Edible use	Medicinal use	Basic plant Sutrop index	Vegetation type^b^	Origin	Management practices	Management site with respect to natural distribution
Amaranthaceae	*Alternanthera caracasana* Kunth	ERL-21, SRL-93	Maravilla			Yes	0	Bal, Sol	Ixcatlán	Forage, gathering, tolerance, uproot	In situ
Amaranthaceae	*Amaranthus hybridus* L.	SRL-79, SRL-80, SRL-1122, SRL-1141, ERL-74, ERL-102	Quelite tintonil		Yes	Yes	0.024	Bal, Sol, TS	Ixcatlán	Enhancement, forage, gathering, protection, tolerance, uproot	In situ
Amaranthaceae	*Chenopodium berlandieri* Moq.	SRL-1139	Quelite de manteca, flor de huizontle		Yes		0.006	Sol	Ixcatlán	Forage, gathering, tolerance	In situ
Amaranthaceae	*Dysphania ambrosioides* (L.) Mosyakin & Clemants	ERL-32, ERL-33, ERL-168, RLF-89, SRL-1136	Epazote		Yes	Yes	0.065	Bal, Sol	Ixcatlán	Gathering, enhancement, protection, propagation, tolerance	In situ
Amaranthaceae	*Gomphrena serrata* L.	RLF-60, RLF-242, SRL-90, SRL-378, SRL-1175	Gallitos			Yes	0	Bal, BEA, BN, Iz, Me, Palm	Ixcatlán	Forage, gathering	In situ
Anacardiaceae	*Actinocheita potentillifolia* (Turcz.) Bullock	RLF-109, RLF-274, SRL-1183, SRL-1368	Tetlate			Yes	0	CaCe, Me, Iz, Palm	Ixcatlán	Gathering	In situ
Anacardiaceae	*Cyrtocarpa procera* Kunth	SRL-1358	Chupandio		Yes		0	CaCe	Ixcatlán	Gathering	In situ
Anacardiaceae	*Pistacia mexicana* Kunth	RLF-326, SRL-1211, SRL-1340, SRL-1523	Socoya			Yes	0	BG, CaCe, Iz, SB, Pal, Sol	Ixcatlán	Forage, gathering, tolerance	In situ
Anacardiaceae	*Rhus chondroloma* Standl.	RLF-282, SRL-1222, SRL-1460	Zumaque		Yes	Yes	0.007	BEA, BEC, Me, Pal, SB, TS	Ixcatlán	Forage, gathering, tolerance	In situ
Anacardiaceae	*Rhus standleyi* F.A.Barkley	RLF-59, RLF-255, SRL-269, SRL-472, SRL-1248, SRL-1470	Encino chaparro, zomaque grueso		Yes	Yes	0.007	BEA, BEC, Iz, Me, Pal, Palm, Sol, TS	Ixcatlán	Gathering, tolerance	In situ
Anacardiaceae	*Rhus virens* Lindl. ex A.Gray	RLF-58, RLF-219, SRL-275, SRL-468, SRL-1218	Zumaque		Yes	Yes	0.007	BEA, BN, Iz, Me, BB , TS	Ixcatlán	Forage, gathering, tolerance	In situ
Apiaceae	*Eryngium bonplandii* F.Delaroche	RLF-6, SRL-132, SRL-384, SRL-1247	Ojo de gallo			Yes	0	BEA, Paz	Ixcatlán	Gathering	In situ
Apiaceae	*Eryngium comosum* F.Delaroche	RLF-127	espinuda			Yes	0	Me	Ixcatlán	Gathering	In situ
Apiaceae	*Eryngium pectinatum* C.Presl ex DC.	RLF-52, SRL-315				Yes	0	BEA, BEC	Ixcatlán	Gathering	In situ
Apocynaceae	*Cascabela thevetia* (L.) Lippold	SRL-1336				Yes	0	CaCe	Ixcatlán	Gathering	In situ
Apocynaceae	*Matelea purpusii* Woodson	SRL-1123	Tecacholo		Yes	Yes	0	BEA, Pal, Sol	Ixcatlán	Gathering, protection, tolerance, propagation	In situ
Apocynaceae	*Plumeria rubra* L.	Photo record	Cacalosuchil	Yes			0	CaMy, Sol	Ixcatlán	Gathering, protection, propagation	Ex situ, in situ
Arecaceae	*Brahea dulcis* (Kunth) Mart.	RLF-155, RLF-191,SRL-462, SRL-463, SRL-1192, SRL-1193	Palma criolla	Yes	Yes	Yes	0.106	BEA, BEC, BG, BN, Iz, Me, Pal, Palm, Sol, TS	Ixcatlán	Enhancement, forage, gathering, protection, tolerance, transplanting of individuals	In situ
Arecaceae	*Brahea dulcis x B. calcarea* Mart. x Liebm.	SRL-1229	Palma media sierra	Yes			0	BEA	Ixcatlán	Gathering, protection	In situ
Aristolochiaceae	*Aristolochia teretiflora* Pfeifer	SRL-1130	Orejita de ratón			Yes	0	Sol, TS	Ixcatlán	Gathering, tolerance, uproot	In situ
Asparagaceae	*Agave kerchovei* Lem.	Photo record	Maguey rabo de león		Yes		0	Iz, Pal	Ixcatlán	Forage, gathering	In situ
Asparagaceae	*Agave potatorum* Zucc.	RLF-285, SRL-403, SRL-1209	Maguey papalomé		Yes	Yes	0.057	BEA, Iz, Me, Pal, Palm, SB, Sol, TS	Ixcatlán	Enhancement, forage, gathering, protection, propagation, tolerance, transplanting of individuals	Ex situ, in situ
Asparagaceae	*Agave salmiana* Otto ex Salm-Dyck subsp. *tehuacanensis* (Karw. ex Salm-Dyck) García-Mend.	Photo record	Maguey cimarrón		Yes	Yes	0	BEA, BN, Pal, Palm, Sol, TS	Ixcatlán	Forage, gathering, protection, tolerance, transplanting of individuals	Ex situ, in situ
Asparagaceae	*Agave scaposa* Gentry	Photo record	Maguey potrero			Yes	0.006	BEM, Sol	Ixcatlán	Gathering, protection, transplanting of individuals	Ex situ, in situ
Asparagaceae	*Agave titanota* Gentry	SRL-404	Maguey tieso		Yes		0	Iz	Ixcatlán	Forage, gathering	In situ
Asparagaceae	*Agave triangularis* Jacobi	SRL-437	Maguey rabo de león, maguey tieso		Yes		0	Iz	Ixcatlán	Forage, gathering	In situ
Asparagaceae	*Agave vivipara* L.	SRL-235, SRL-1353, SRL-1389	Maguey espadín			Yes	0	CaCe, Iz, Pal, SB, Sol, Ts	Ixcatlán	Gathering, protection, propagation	Ex situ, in situ
Asparagaceae	*Beaucarnea stricta* Lem.	RLF-149	Sotol	Yes			0.005	Iz	Ixcatlán	Gathering, protection	In situ
Asparagaceae	*Dasylirion serratifolium* (Karw. ex Schult. & Schult.f.) Zucc.	RLF-156, SRL-420, SRL-1473, SRL-1521	Cucharilla, manita	Yes	Yes		0.010	BG, Me	Ixcatlán	Forage, gathering	In situ
Asparagaceae	*Echeandia paniculata* Rose	SRL-442, SRL-1114	Cebolla de cacalote		Yes	Yes	0	BEA, Iz, Me	Ixcatlán	Gathering	In situ
Asparagaceae	*Nolina longifolia* (Karw. ex Schult. & Schult.f.) Hemsl.	SRL-228	Sotol	Yes			0	BEA, Me	Ixcatlán	Gathering	In situ
Asparagaceae	*Yucca periculosa* Baker	SRL-1505	Tohuizote		Yes		0	AA	Ixcatlán	Gathering	In situ
Bignoniaceae	*Tecoma stans* (L.) Juss. ex Kunth	RLF-13, RLF-56, RLF-249, SRL-438, SRL-465, SRL-1307	Tronadora			Yes	0	BEA, BN, Iz, Me	Ixcatlán	Forage, gathering	In situ
Boraginaceae	*Antiphytum caespitosum* I.M.Johnst.	RLF-125, SRL-99, SRL-1400, SRL-1466	Semonilla			Yes	0	BN, Me, Palm	Ixcatlán	Gathering	In situ
Brassicaceae	*Brassica rapa* L.	SRL-1536	Mostaza		Yes		0	Bal, Sol, TS	Naturalized, from other continents	Gathering, tolerance, uproot	Ex situ
Brassicaceae	*Capsella bursa-pastoris* (L.) Medik.	SRl-182, SRL-1324	Lentejilla			Yes	0	Bal, Sol	Naturalized, from other continents	Gathering, tolerance, uproot	Ex situ
Brassicaceae	*Descurainia virletii* (E.Fourn.) O.E.Schulz	SRL-35	Mostaza		Yes		0	Bal, Sol	Ixcatlán	Forage, gathering	In situ
Brassicaceae	*Eruca vesicaria* (L.) Cav.	RLF-309, SRL-39, SRL-1131	Jaramón			Yes	0	Bal, Sol, TS	Naturalized, from other continents	Forage, gathering, tolerance, uproot	Ex situ
Brassicaceae	*Lepidium virginicum* L.	ERL-109, RLF-70, RLF-103, RLF-179, SRL-1320	Lentejilla			Yes	0	Bal, BEA, Sol	Ixcatlán	Forage, gathering, protection, tolerance	In situ
Brassicaceae	*Nasturtium officinale* R.Br.	SRL-199	Berro		Yes		0.002	VR	Naturalized, from other continents	Gathering	In situ
Bromeliaceae	*Catopsis compacta* Mez	RLF-335, SRL1253	Soluche de jarrita	Yes		Yes	0	BEA, Iz, Sol	Ixcatlán	Gathering, protection, transplanting of individuals	Ex situ, in situ
Bromeliaceae	*Tillandsia acyrostachys* E.Morren ex Baker	SRL-1492		Yes			0	Me	Ixcatlán	Gathering, transplanting of individuals	In situ
Bromeliaceae	*Tillandsia bourgaei* Baker	SRL-1197	Soluche blanco		Yes		0	BEA	Ixcatlán	Gathering	In situ
Bromeliaceae	*Tillandsia grandis* Schltdl.	SRL-1472	Jarrilla	Yes			0	CaCe, Me, Sol	Ixcatlán	Gathering, protection, transplanting of individuals	Ex situ, in situ
Bromeliaceae	*Tillandsia gymnobotrya* Baker	SRL-1201, SRL-1435	Soluche blanco, soluche de flor colorada	Yes	Yes	Yes	0	BEM	Ixcatlán	Gathering	In situ
Bromeliaceae	*Tillandsia juncea* (Ruiz & Pav.) Poir.	RLF-81, SRL-1246, SRL-1254	Soluche	Yes			0	BEA, Sol	Ixcatlán	Gathering	In situ
Bromeliaceae	*Tillandsia macdougallii* L.B.Sm.	RLF-84, SRL-224, SRL-1242, SRL-1250	Soluche		Yes		0	BEA, Pal, Sol, VR	Ixcatlán	Gathering, protection, transplanting of individuals	Ex situ, in situ
Bromeliaceae	*Tillandsia recurvata* (L.) L.	SRL-211	Soluchito			Yes	0	Palm, Sol	Ixcatlán	Gathering, tolerance	In situ
Bromeliaceae	*Tillandsia* sp.	SRL-1252	Soluche cimarrón, soluche ixtludo	Yes			0	BEA, Pal	Ixcatlán	Gathering, protection, transplanting of individuals	Ex situ, in situ
Bromeliaceae	*Tillandsia* sp.	SRL-1243	Soluche	Yes	Yes		0	BEA	Ixcatlán	Gathering	In situ
Bromeliaceae	*Tillandsia usneoides* (L.) L.	SRL-138, SRL-1245	Apasle	Yes			0	BEA, BEM, Pal, Sol	Ixcatlán	Gathering, protection, propagation, transplanting of individuals	Ex situ, in situ
Buddlejaceae	*Buddleja parviflora* Kunth	ERL-197, SRL-371, SRL-1207, SRL-1522	Lengua de vaca, tepozán			Yes	0	BEA, BG, Palm, Sol	Ixcatlán	Gathering, tolerance	In situ
Burseraceae	*Bursera biflora* (Rose) Standl.	RJS-11, RLF-122, SRL-1219	Copal colorado, copal amarillo, copal criollo	Yes		Yes	0	Iz, Me, SB	Ixcatlán	Gathering, protection, propagation, transplanting of individuals	Ex situ, in situ
Burseraceae	*Bursera fagaroides* (Kunth) Engl.	SRL-349	Copalillo	Yes			0	Me	Ixcatlán	Forage, gathering	In situ
Burseraceae	*Bursera pontiveteris* Rzed., Calderón & Medina	SRL-1271	Copalillo blanco	Yes			0	Me	Ixcatlán	Gathering, protection	In situ
Burseraceae	*Bursera schlechtendalii* Engl.	SRL-1367	Aceitillo			Yes	0	CaCe	Ixcatlán	Forage, gathering	In situ
Cactaceae	*Ferocactus recurvus* (Mill.) Borg	SRL-1419	Bizniaga grande		Yes		0	Paz, Sol, TS	Ixcatlán	Forage, gathering, protection, tolerance, transplanting of individuals	Ex situ, in situ
Cactaceae	*Ferocactus macrodiscus* (Mart.) Britton & Rose	SRL-402	Bizniaga		Yes		0	Paz, Sol	Ixcatlán	Forage, gathering, protection, transplanting of individuals	Ex situ, in situ
Cactaceae	*Mammillaria haageana* Pfeiff.	SRL-387, SRL-1480	Bizniaga chiquita	Yes	Yes		0	BEA, Iz, Me, Palm, Sol	Ixcatlán	Gathering, protection, transplanting of individuals	Ex situ, in situ
Cactaceae	*Mammillaria sphacelata* Mart.	Photo record	Biznaga	Yes	Yes		0	BEA, BN, Me, Pal, Sol, TS	Ixcatlán	Gathering, protection, tolerance, transplanting of individuals	Ex situ, in situ
Cactaceae	*Opuntia depressa* Rose	SRL-238	Nopal de coyote			Yes	0	BEA,TS	Ixcatlán	Forage, gathering, tolerance	In situ
Cactaceae	*Opuntia lasiacantha* Pfeiff.	SRL-477	Nopal pachón		Yes		0.042	Sol, TS	Ixcatlán	Gathering, protection, propagation, tolerance, transplanting of individuals	Ex situ, in situ
Cactaceae	*Opuntia* sp.	Photo record	Nopal de coyote, nopal tuna roja		Yes		0	Palm, Sol	Ixcatlán	Gathering, forage, propagation, tolerance, transplanting of individuals	Ex situ, in situ
Cannabaceae	*Celtis caudata* Planch.	ERL-79, ERL-155, ERL-194, ERL-222, SRL-1475	Malintze, moralillo		Yes		0	Me, Sol	Ixcatlán	Gathering, protection, tolerance	In situ
Chenopodiaceae	*Chenopodium murale* L.	RLF-184, SRL-194, SRL-1121, SRL-1140, SRL-1321	Quelite de guajolote		Yes	Yes	0	Bal, Sol	Naturalized, from other continents	Forage, gathering, tolerance, uproot	Ex situ
Commelinaceae	*Tradescantia crassifolia* Cav.	SRL-149				Yes	0	Me	Ixcatlán	Gathering	In situ
Compositae	*Ageratina espinosarum* (A.Gray) R.M.King & H.Rob.	RLF-36, SRL-114, SRL-291, SRL-325, SRL-363, SRL-1279				Yes	0	BEA, BEC, BG, BN, Iz, Me, Pal, Palm, Sol, TS	Ixcatlán	Forage, gathering, tolerance, uproot	In situ
Compositae	*Ageratina mairetiana* (DC.) R.M.King & H.Rob.	SRL-186, SRL-390	Hierba de ángel			Yes	0.014	BEA, Pal, Sol	Ixcatlán	Forage, gathering, protection, tolerance, transplanting of individuals	Ex situ, in situ
Compositae	*Ageratina* sp.	RLF-116, SRL-74	Hierba de ángel			Yes	0	Me, Sol	Ixcatlán	Forage, gathering	In situ
Compositae	*Ageratina* sp.	SRL-208	Oreganillo			Yes	0	Pal, Sol	Ixcatlán	Gathering, tolerance	In situ
Compositae	*Ambrosia psilostachya* DC.	RLF-9				Yes	0	BEA, BN, Me, Paz	Ixcatlán	Gathering	In situ
Compositae	*Baccharis salicina* Torr. & A.Gray	SRL-1151	Chamizo			Yes	0	BEA	Ixcatlán	Gathering	In situ
Compositae	*Barkleyanthus salicifolius* (Kunth) H.Rob. & Brettell	SRL-190, SRL-1531, ERL-27, ERL-83, ERL-190, ERL-218	Somiate			Yes	0.003	BG, Pal, Palm, Sol	Ixcatlán	Forage, gathering, tolerance, transplanting of individuals	In situ
Compositae	*Bidens pilosa* L.	SRL-4, SRL-1285	Oaxaqueña			Yes	0	BG, Pal, Sol, TS	Ixcatlán	Forage, gathering, tolerance, uproot	In situ
Compositae	*Brickellia veronicifolia* (Kunth) A.Gray	RLF-11, RLF-203, RLF-206, SRL-293, SRL-361, SRL-1276, ERL-101	Oreganillo, orejita de ratón			Yes	0	BEA, BN, Iz, Me, Pal, Palm, Sol, TS	Ixcatlán	Forage, gathering, tolerance	In situ
Compositae	*Chrysactinia mexicana* A.Gray	RLF-154, SRL-1163	Hierba de San Nicolás			Yes	0	Palm	Ixcatlán	Gathering, protection	In situ
Compositae	*Cirsium mexicanum* DC.	SRL-435	Lechuga cimarrón		Yes		0	BG, Pal	Ixcatlán	Forage, gathering	In situ
Compositae	*Cosmos bipinnatus* Cav.	ERL-4, ERL-166, ERL-167, SRL-45, SRL-47	Jazmín	Yes			0	Sol	Naturalized-from other parts of Mexico	Gathering, enhancement, protection, propagation, tolerance	Ex situ
Compositae	*Dahlia apiculata* (Sherff) P.D.Sorensen	RLF-259, SRL-91, SRL-1199, ERL-133, ERL-148	Dalia corriente, ticurrichi	Yes			0	BEA, BEM, Pal, Sol	Ixcatlán	Gathering, protection, propagation, transplanting of individuals	Ex situ, in situ
Compositae	*Dahlia coccinea* Cav.	RLF-96, RLF-260, SRL-423, SRL-1160, SRL-1186	Dalia	Yes			0	BEA, BEM, BG, Me, Pal, Sol	Ixcatlán	Gathering, protection, propagation, transplanting of individuals	Ex situ, in situ
Compositae	*Gnaphalium* sp.	SRL-297				Yes	0	Paz	Ixcatlán	Gathering	In situ
Compositae	*Grindelia inuloides* Willd.	RLF-14, SRL-66, SRL-107, SRL-278, SRL-295, SRL-365, SRL-1547	Árnica			Yes	0.029	BEA, BN, Pal, Palm, Paz, Sol	Ixcatlán	Enhancement, gathering, protection, propagation, transplanting of individuals	Ex situ, in situ
Compositae	*Gymnosperma glutinosum* (Spreng.) Less.	RLF-72, RLF-121, SRL-75, SRL-290, SRL-1117, SRL-1287, ERL-25	Cerilla, popote			Yes	0.002	Bal, BEA, BN, Iz, Me, Pal, Palm, Sol, TS	Ixcatlán	Forage, gathering, tolerance, uproot	In situ
Compositae	*Helenium mexicanum* Kunth	RLF-25, SRL-1116, SRL-1134	Chiche de perro			Yes	0	BEA, Palm	Ixcatlán	Gathering	In situ
Compositae	*Montanoa tomentosa* Cerv.	RLF-300, SRL-2	Oaxaqueña			Yes	0	Iz, Sol	Ixcatlán	Gathering	In situ
Compositae	*Neurolaena lobata* (L.) R.Br. ex Cass.	SRL-198	Naranjillo	Yes		Yes	0	VR	Ixcatlán	Gathering	In situ
Compositae	*Parthenium bipinnatifidum* (Ortega) Rollins	ERL-9, RLF-87, RLF-178, SRL-34, SRL-82, SRL-445, SRL-1325	Hierba cenizo			Yes	0	Sol	Ixcatlán	Forage, gathering, tolerance, uproot	In situ
Compositae	*Parthenium tomentosum* DC.	SRL-1213, SRL-1375	Palo prieto			Yes	0	CaCe, SB	Ixcatlán	Gathering	In situ
Compositae	*Perymenium* sp.	RLF-251	Cahual			Yes	0	Iz	Ixcatlán	Forage, gathering	In situ
Compositae	*Pinaropappus roseus* (Less.) Less.	RJS-8, SRL-407, SRL-1526	Chipule			Yes	0	Bal, BG, Iz, Paz	Ixcatlán	Gathering	In situ
Compositae	*Piqueria trinervia* Cav.	RLF-8				Yes	0	BEA	Ixcatlán	Forage, gathering	In situ
Compositae	*Porophyllum linaria* (Cav.) DC.	RLF-18, SRL-158, SRL-357, SRL-1150, ERL-141	Pepitza		Yes	Yes	0	BEA, BN, Me, Palm, Paz, Sol, TS	Ixcatlán	Gathering, protection, propagation, tolerance, transplanting of individuals	Ex situ, in situ
Compositae	*Porophyllum ruderale* subsp. *macrocephalum* (DC.) R.R.Johnson	RLF-318, SRL-1539	Papaloquelite		Yes		0.004	Me, Sol	Ixcatlán	Enhancement, gathering, protection, propagation, tolerance, transplanting of individuals	Ex situ, in situ
Compositae	*Psacalium paucicapitatum* (B.L.Rob. & Greenm.) H.Rob. & Brettell	RLF-193, SRL-1159	Hierba de camote de venado			Yes	0	BEA, Iz	Ixcatlán	Gathering, protection, transplanting of individuals	Ex situ, In situ
Compositae	*Roldana ehrenbergiana* (Klatt) H.Rob. & Brettell	SRL-1152	Hierba de perro			Yes	0	BEA	Ixcatlán	Gathering	In situ
Compositae	*Sanvitalia procumbens* Lam.	RLF-42, SRL-12, SRL-1179	Ojo de gallo			Yes	0	Me, Palm, Sol, TS	Ixcatlán	Forage, gathering, tolerance, uproot	In situ
Compositae	*Senecio praecox* (Cav.) DC.	ERL-191, SRL-1487	Consuelda, pata de león			Yes	0	Me, Sol	Ixcatlán	Gathering, protection, transplanting of individuals	Ex situ, in situ
Compositae	*Sonchus oleraceus* (L.) L.	ERL-10, SRL-1126	Chicoria			Yes	0	Sol	Naturalized, from other continents	Gathering, tolerance, uproot	Ex situ
Compositae	*Stevia lucida* Lag.	SRL-332, SRL-339	Chamalacate	Yes			0	BN, Iz, Me, Palm, TS	Ixcatlán	Gathering, forage, tolerance, uproot	In situ
Compositae	*Stevia* sp.	RLF-170, RLF-183, SRL-32, SRL-97, SRL-1281	Cahual delgado			Yes	0	BN, Pal, Sol, TS	Ixcatlán	Forage, gathering, tolerance	In situ
Compositae	*Stevia* sp.	RLF-276	Cahual prieto			Yes	0	BEA, Pal	Ixcatlán	Gathering	In situ
Compositae	*Stevia* sp.	SRL-1262		Yes		Yes	0	Me	Ixcatlán	Gathering	In situ
Compositae	*Stevia* sp.	SRL-1295				Yes	0	Pal	Ixcatlán	Forage, gathering	In situ
Compositae	*Stevia caracasana* DC.	RLF-211, SRL-1289, SRL-1293, SRL-1402		Yes			0	Iz, Pal, Palm	Ixcatlán	Gathering, tolerance	In situ
Compositae	*Tagetes erecta* L.	ERL-12, ERL-62, ERL-117, ERL-118, ERL-134, ERL-149, ERL-151, ERL-152, ERL-159, SRL-7, SRL-408, SRL-1142	Cempasuchí	Yes			0.003	Sol, TS	Naturalized-from other parts of Mexico	Enhancement, protection, propagation, tolerance, transplanting of individuals	Ex situ
Compositae	*Tagetes lucida* Cav.	RLF-3, SRL-377, SRL-1232, SRL-1426	Pericón	Yes	Yes	Yes	0.003	BEA, Paz	Ixcatlán	Gathering	In situ
Compositae	*Tagetes lunulata* Ortega	ERL-137	Cempasuchí chiquito	Yes			0	Sol	Ixcatlán	Gathering, enhancement, forage, protection, tolerance	In situ
Compositae	*Taraxacum campylodes* G.E.Haglund	ERL-106, SRL-89	Achicoria		Yes	Yes	0	Sol	Naturalized, from other continents	Gathering, tolerance, uproot	Ex situ
Compositae	*Tridax coronopifolia* (Kunth) Hemsl.	SRL-104				Yes	0	BN	Ixcatlán	Gathering	In situ
Compositae	*Viguiera dentata* (Cav.) Spreng.	RLF-227, SRL-164, SRL-1277, SRL-1302	Chimalacate	Yes	Yes	Yes	0	BEA, BEC, BG, Iz, Me, Pal, Sol, TS	Ixcatlán	Forage, gathering, tolerance, uproot	In situ
Compositae	*Viguiera grammatoglossa* DC.	RLF-233, RLF-298, SRL-347, SRL-1286	Cahual prieto			Yes	0	BG, Iz, Me, Pal, Palm, TS	Ixcatlán	Forage, gathering, tolerance, uproot	In situ
Compositae	*Zinnia peruviana* (L.) L.	RLF-12, RLF-234, SRL-367, SRL-1173, SRL-1261, SRL-1317	Gallito	Yes		Yes	0	BEA, BN, Iz, Me, Palm, TS	Ixcatlán	Forage, gathering, tolerance, uproot	In situ
Compositae		SRL-1214	Jazmincillo, cahual blanco	Yes			0	SB	Ixcatlán	Gathering	In situ
Compositae		SRL-1372				Yes	0	CaCe	Ixcatlán	Gathering	In situ
Compositae		ERL-121, SRL-1275	Cahual prieto			Yes	0	Pal, Sol, VR	Ixcatlán	Gathering, tolerance	In situ
Compositae		SRL-1478	Hierba de ángel, oaxaqueña			Yes	0	BEA	Ixcatlán	Gathering	In situ
Compositae		SRL-1339	Cempasuchí de molito de campo			Yes	0	CaCe	Ixcatlán	Gathering	In situ
Convolvulaceae	*Dichondra argentea* Humb. & Bonpl. ex Wild.	RLF-71, SRL-134, SRL-167	Orejita de ratón			Yes	0	BEA, BEC, BN, Me, Palm	Ixcatlán	Gathering	In situ
Convolvulaceae	*Ipomoea aff. populina* House	SRL-1306	Jícama		Yes		0	Me	Ixcatlán	Forage, gathering	In situ
Convolvulaceae	*Ipomoea conzattii* Greenm.	SRL-1491, SRL-1510	Jícama de cerro		Yes		0	CaMy, Me	Ixcatlán	Forage, gathering	In situ
Convolvulaceae	*Ipomoea purpurea* (L.) Roth	ERL-14, RLF-44, RLF-45, SRL-145, SRL-448	Quiebra platos			Yes	0	BEA, Me, Paz, Sol, TS	Ixcatlán	Gathering, tolerance, uproot	In situ
Crassulaceae	*Echeveria gigantea* Rose & Purpus	SRL-1313	Siempreviva grande, lengua de vaca, oreja de toro			Yes	0	MR, Sol	Ixcatlán	Gathering, protection, transplanting of individuals	Ex situ, in situ
Crassulaceae	*Echeveria nodulosa* (Baker) Otto	SRL-356, SRL-1187, SRL-1255, SRL-1436	Siempreviva chiquita			Yes	0	BEA, Me, Iz, Palm, Sol	Ixcatlán	Gathering, protection, transplanting of individuals	Ex situ, in situ
Crassulaceae	*Sedum dendroideum* Moc. & Sessé ex DC.	SRL-77, SRL-195, ERL-97, ERL-174	Siempreviva	Yes		Yes	0	NE, Sol	Ixcatlán	Gathering, protection, propagation, transplanting of individuals	Ex situ, in situ
Cucurbitaceae	*Cucurbita pedatifolia* L.H.Bailey	ERL-120, RLF-268, SRL-1135	Calabacita amarga			Yes	0	Bal, Pal, Sol	Ixcatlán	Forage, gathering, tolerance, uproot	In situ
Cucurbitaceae	*Cyclanthera dissecta* (Torr. & A.Gray) Arn.	SRL-151	Chayotito			Yes	0	Me, TS	Ixcatlán	Forage, gathering, tolerance, uproot	In situ
Cucurbitaceae	*Schizocarpum filiforme* Schrad.	SRL-1260	Chayotito			Yes	0	Sol, TS	Ixcatlán	Forage, gathering, tolerance, uproot	In situ
Cucurbitaceae	*Sicyos laciniatus* L.	ERL-100, RLF-90, SRL-14	Chayotillo, pegajosa			Yes	0	Sol, TS	Ixcatlán	Forage, gathering, tolerance, uproot	In situ
Cupressaceae	*Juniperus flaccida* Schltdl.	ERL-187, RLF-126, RLF-134, SRL-123, SRL-412, SRL-1119	Nebro	Yes		Yes	0.053	BEA, BEC, BG, BN, Iz, Me, Pal, Palm, Sol, TS	Ixcatlán	Forage, gathering, protection, tolerance, transplanting of individuals	In situ
Cupressaceae	*Taxodium huegelii* C.Lawson	SRL-210, SRL-434, SRL-1294	Sabino	Yes			0.004	BG, Pal, Palm, Sol	Ixcatlán	Gathering, protection, propagation, tolerance, transplanting of individuals	Ex situ, in situ
Cyperaceae	*Carex* sp.	RLF-133	Pasto			Yes	0	Me	Ixcatlán	Forage, gathering	In situ
Cyperaceae	*Eleocharis acicularis* (L.) Roem. & Schult.	RLF-138	Pasto de arroyo			Yes	0	VR	Ixcatlán	Gathering	In situ
Ebenaceae	*Diospyros oaxacana* Standl.	SRL-1446	Zapotito		Yes		0	VR	Ixcatlán	Forage, gathering	In situ
Ericaceae	*Arbutus xalapensis* Kunth	ERL-172, RLF-124, RLF-279, SRL-1477	Madroño, ollita	Yes		Yes	0.018	BEA, BEC, BEM, BN, Me, TS	Ixcatlán	Gathering, tolerance	In situ
Euphorbiaceae	*Cnidosculus tehuacanensis* Breckon	Photo record	Mala mujer			Yes	0	Iz, Palm	Ixcatlán	Gathering	In situ
Euphorbiaceae	*Euphorbia dioeca* Kunth	ERL-107, RLF-7, SRL-359	Celedonia			Yes	0	BEA, Sol	Ixcatlán	Gathering, tolerance, uproot	In situ
Euphorbiaceae	*Euphorbia macropus* (Klotzsch & Garcke) Boiss.	SRL-1120	Hierba de chicle		Yes	Yes	0	Palm	Ixcatlán	Gathering	In situ
Euphorbiaceae	*Jatropha neopauciflora* Pax	SRL-1357	Sangre de grado, aceitillo			Yes	0	CaCe	Ixcatlán	Forage, gathering	In situ
Euphorbiaceae	*Ricinus communis* L.	ERL-116, ERL-144, ERL-145, ERL-243, SRL-23, SRL-1129	Gría			Yes	0	Bal, Sol	Naturalized, from other continents	Enhancement, gathering, protection, tolerance, transplanting of individuals, uproot	In situ
Fagaceae	*Quercus acutifolia* Née	SRL-1226, SRL-1516	Encino colorado	Yes		Yes	0.072	BEM	Ixcatlán	Forage, gathering, protection, transplanting of individuals, propagation	Ex situ, in situ
Fagaceae	*Quercus castanea* Née	RLF-78, SRL-1233, SRL-1408, SRL-1425, SRL-1431	Encino prieto, encino blanco	Yes		Yes	0.056	BEA, BEM, BN, TS	Ixcatlán	Forage, gathering, tolerance	In situ
Fagaceae	*Quercus conspersa* Benth.	SRL-1156	Encino colorado	Yes		Yes	0.072	BEM	Ixcatlán	Forage, gathering, protection	In situ
Fagaceae	*Quercus laeta* Liebm.	RLF-68, SRL-143, SRL-253, SRL-385, SRL-1230	Encino prieto, encino amarillo	Yes			0.140	BEA, BEC, Pal, Sol	Ixcatlán	Forage, gathering, protection, propagation, tolerance	Ex situ, in situ
Fagaceae	*Quercus liebmannii* Oerst. ex Trel.	SRL-1107, SRL-1514	Encino amarillo	Yes			0.140	BEA, Me, Palm, TS	Ixcatlán	Forage, gathering, protection, propagation, tolerance, transplanting of individuals	Ex situ, in situ
Fagaceae	*Quercus obtusata* Bonpl.	SRL-1423	Encino prieto	Yes			0.056	BEM	Ixcatlán	Forage, gathering, protection	In situ
Fagaceae	*Quercus polymorpha* Schltdl. & Cham.	SRL-1503	Encino prieto	Yes			0	BG, Pal	Ixcatlán	Forage, gathering, protection	In situ
Fagaceae	*Quercus urbanii* Trel	RLF-161, SRL-252, SRL-475, SRL-1228	Encino cucharilla	Yes			0.074	BEA, BEC, TS	Ixcatlán	Forage, gathering, protection, tolerance	In situ
Iridaceae	*Tigridia pavonia* (L.f.) DC.	RLF-201		Yes			0	Iz	Ixcatlán	Gathering	In situ
Krameriaceae	*Krameria cytisoides* Cav.	RLF-97, SRL-251, SRL-1265, SRL-1376	Chayotillo de burro, borreguito			Yes	0	Me, Palm	Ixcatlán	Forage, gathering	In situ
Lamiaceae	*Clinopodium mexicanum* (Benth.) Govaerts	RLF-131, RLF-262, SRL-1190, SRL-1280, SRL-1403	Chipito		Yes	Yes	0	BEA, Me, Pal, Sol, VR	Ixcatlán	Gathering, protection, transplanting of individuals	Ex situ, in situ
Lamiaceae	*Hyptis* sp.	SRL-209				Yes	0	Sol	Ixcatlán	Gathering, tolerance	In situ
Lamiaceae	*Leonotis nepetifolia* (L.) R.Br.	SRL-1315		Yes			0	Sol	Naturalized, from other continents	Gathering, enhancement, protection, propagation, tolerance	Ex situ
Lamiaceae	*Marrubium vulgare* L.	ERL-80, RLF-64, SRL-29, SRL-1146	Manrrubio			Yes	0	Bal, Pal, Sol	Naturalized, from other continents	Gathering, tolerance, uproot	In situ
Lamiaceae	*Salvia candicans* M.Martens & Galeotti	SRL-155, SRL-1456				Yes	0	Me	Ixcatlán	Gathering	In situ
Lamiaceae	*Salvia oaxacana* Fernald	RLF-232, SRL-1161, SRL-1188	Mirto cimarrón			Yes	0	BEA	Ixcatlán	Forage, gathering	In situ
Lamiaceae	*Salvia purpurea* Cav.	RLF-1, RLF-194, SRL-116, SRL-273, SRL-1195, SRL-1202, SRL-1397, SRL-1420	Terciopelo	Yes		Yes	0	BEA, BEC, BN, Iz	Ixcatlán	Gathering	In situ
Lamiaceae	*Salvia sessei* Benth.	RLF-33, RLF-195, SRL-1162	Oaxaqueña			Yes	0	BEA, BEM	Ixcatlán	Gathering	In situ
Lamiaceae	*Salvia* sp.	Photo record	Mirto			Yes	0	Sol	Ixcatlán	Gathering, tolerance	In situ
Lamiaceae	*Salvia* sp.	SRL-140	Marrubio macho			Yes	0	BEA	Ixcatlán	Gathering	In situ
Lamiaceae	*Salvia thymoides* Benth.	RLF-245, SRL-1469	Oreganillo cenizo			Yes	0	Iz, Me	Ixcatlán	Gathering	In situ
Lamiaceae	*Salvia tiliifolia* Vahl	ERL-28-ERL-112, RLF-162, SRL-3	Chía			Yes	0	Bal, Sol, TS	Ixcatlán	Gathering, tolerance, uproot	In situ
Lamiaceae	*Salvia circinnata* Cav.	RLF-215, SRL-1291				Yes	0	Iz, Palm	Ixcatlán	Gathering	In situ
Lauraceae	*Litsea glaucescens* Kunth	SRL-1157, SRL-1515	Laurel	Yes	Yes		0.010	BEA	Ixcatlán	Gathering, propagation	In situ
Leguminosae	*Calliandra* sp.	SRL-276	Guaje de gamito		Yes		0	BEA, BEC, BG, BN, Me	Ixcatlán	Forage, gathering	In situ
Leguminosae	*Crotalaria pumila* Ortega	SRL-103, SRL-364				Yes	0	BN, Palm	Ixcatlán	Forage, gathering	In situ
Leguminosae	*Dalea carthagenensis* (Jacq.) J.F.Macbr.	RLF-115, RLF-168, RLF-222, SRL-154, SRL-417, SRL-1185, SRL-1299	Hierba de Obo			Yes	0	BG, Iz, Me, TS	Ixcatlán	Forage, gathering, tolerance, uproot	In situ
Leguminosae	*Dalea* sp.	SRL-348			Yes		0	Me	Ixcatlán	Gathering	In situ
Leguminosae	*Dalea tomentosa* (Cav.) Willd.	RLF-214, SRL-214				Yes	0	BN, Iz, Palm	Ixcatlán	Forage, gathering	In situ
Leguminosae	*Desmanthus* sp.	RLF-225	Tepeguaje cimarrón		Yes		0	Iz	Ixcatlán	Forage, gathering	In situ
Leguminosae	*Desmanthus virgatus* (L.) Willd.	SRL-368	Guajito de gabito		Yes		0	Palm	Ixcatlán	Gathering	In situ
Leguminosae	*Leucaena* sp.	SRL-1158	Guaje de gamito		Yes		0	BEA	Ixcatlán	Gathering	In situ
Leguminosae	*Lupinus leptophyllus* Cham. & Schltdl.	SRL-1410		Yes			0	BEA	Ixcatlán	Gathering	In situ
Leguminosae	*Phaseolus* sp.	SRL-1206	Ejote de venado		Yes		0	BEA	Ixcatlán	Forage, gathering	In situ
Leguminosae	*Piscidia grandifolia* (Donn.Sm.) I.M.Johnst.	SRL-1210				Yes	0	SB	Ixcatlán	Gathering	In situ
Leguminosae	*Prosopis laevigata* (Willd.) M.C.Johnst.	SRL-1388	Mezquite		Yes	Yes	0	Pal, SB, Sol	Ixcatlán	Forage, gathering, tolerance	In situ
Leguminosae	*Senna guatemalensis* (Donn.Sm.) H.S.Irwin & Barneby	RLF-246, RLF-295		Yes		Yes	0	Iz	Ixcatlán	Forage, gathering	In situ
Leguminosae	*Trifolium* sp.	SRL-375				Yes	0	BEA	Naturalized, unknown origin	Forage, gathering	Ex situ
Leguminosae	*Zornia reticulata* Sm.	SRL-300				Yes	0	Paz	Ixcatlán	Forage, gathering	In situ
Leguminosae		SRL-1212	Tepeguaje			Yes	0	SB	Ixcatlán	Forage, gathering	In situ
Leguminosae		SRL-1217				Yes	0	SB	Ixcatlán	Forage, gathering	In situ
Linaceae	*Linum* sp.	RLF-175				Yes	0	Palm, TS	Ixcatlán	Forage, gathering, tolerance, uproot	In situ
Loasaceae	*Mentzelia hispida* Willd.	RLF-54, RLF-94, SRL-428	Pegajosa			Yes	0	Bal, BEA, BG	Ixcatlán	Gathering, tolerance, uproot	In situ
Lythraceae	*Cuphea* sp.	RLF-100, RLF-143, RLF-172, SRL-20, SRL-350, SRL-1178		Yes		Yes	0	Me, Sol, Palm, TS	Ixcatlán	Forage, gathering, tolerance, uproot	In situ
Lythraceae	*Cuphea* sp.	SRL-25		Yes			0	BN, Palm, Sol	Ixcatlán	Gathering	In situ
Lythraceae	*Cuphea* sp.	SRL-105, SRL-296		Yes			0	BEA, BN, Paz	Ixcatlán	Gathering	In situ
Malpighiaceae	*Bunchosia* sp.	SRL-451	Huevo de gato		Yes		0	Sol	Ixcatlán	Gathering, tolerance	In situ
Malpighiaceae	*Galphimia multicaulis* A.Juss.	RLF-65, RLF-293, SRL-1177	Flor de chivo	Yes			0	BEA, BEC, Iz, Me, Palm	Ixcatlán	Forage, gathering	In situ
Malpighiaceae	*Gaudichaudia galeottiana* (Nied.) Chodat	RLF-241				Yes	0	Iz	Ixcatlán	Gathering	In situ
Malpighiaceae	*Malpighia galeottiana* A.Juss.	SRL-362, SRL-471, SRL-1272	Nanche		Yes		0	Me, Palm, TS	Ixcatlán	Forage, gathering, tolerance	In situ
Malvaceae	*Anoda cristata* (L.) Schltdl.	RLF-67, RLF-277, SRL-6, SRL-446, SRL-1125	Quelite de malva, violeta		Yes	Yes	0	Bal, BEA, Pal, Sol, TS	Ixcatlán	Forage, gathering, tolerance, uproot	In situ
Malvaceae	*Malva parviflora* L.	ERL-30, ERL-90, SRL-205, SRL-1124, SRL-1143	Malva			Yes	0	Bal, Sol, TS	Naturalized, from other continents	Enhancement, forage, gathering, tolerance, uproot	In situ
Martyniaceae	*Proboscidea louisianica* (Mill.) Thell.	SRL-1318	Cuerno de toro		Yes		0	Bal, Palm, Sol, TS	Ixcatlán	Gathering, tolerance	In situ
Meteoriaceae	*Meteorium deppei* (Hornsch. ex Müll. Hal.) Mitt.	SRL-1432	Musgo	Yes			0	BEA, BM, Sol	Ixcatlán	Gathering, protection, transplanting of individuals	Ex situ, in situ
Moraceae	*Ficus crocata* (Miq.) Mart. ex Miq.	SRL-76, SRL-1171	Amate		Yes		0.006	Sol	Ixcatlán	Gathering, tolerance	In situ
Moraceae	*Morus celtidifolia* Kunth	ERL-55, ERL-78, ERL-55, ERL-78, ERL-124, ERL-128, ERL-129, ERL-214, ERL-220, ERL-221, SRL-55, SRL-1517	Moral, morera		Yes		0.051	AA, Sol	Ixcatlán	Gathering, protection, tolerance	In situ
Nyctaginaceae	*Mirabilis jalapa* L.	ERL-29, ERL-99, SRL-11, SRL-421, SRL-1145	Hierba cuchi, maravilla			Yes	0.003	Bal, BG, Sol	Ixcatlán	Forage, gathering, tolerance, uproot	In situ
Onagraceae	*Gaura coccinea* Nutt. ex Pursh	SRl-17, SRL-411	Gradiolita			Yes	0	Bal, Sol	Ixcatlán	Forage, gathering, tolerance, uproot	In situ
Onagraceae	*Oenothera pubescens* Willd. ex Spreng.	RLF-76, RLF-113, SRL-22, SRL-40, SRL-150, SRL-213	Campanita grande			Yes	0	Bal, BEA, Me, Sol	Ixcatlán	Gathering, tolerance, uproot	In situ
Onagraceae	*Oenothera rosea* L’Her. ex Aiton	SRL-1127, SRL-1322	Sanguinaria			Yes	0	Bal, Sol	Ixcatlán	Gathering, tolerance, uproot	In situ
Orchidaceae	*Barkeria lindleyana* subsp. *vanneriana* (Rchb.f.) Thien	SRL-1509	Monjita de peña	Yes			0	CaMy	Ixcatlán	Gathering, protection, transplanting of individuals	Ex situ, in situ
Orchidaceae	*Dichromanthus cinnabarinus* (Lex.) Garay	RLF-223, RLF-289, SRL-1155, SRL-1172	Cola de león	Yes		Yes	0	BEA, Iz, Palm	Ixcatlán	Gathering	In situ
Orchidaceae	*Encyclia hanburyi* (Lindl.) Schltr.	SRL-1519	Monjita morada de campo	Yes			0	Me, Sol	Ixcatlán	Gathering, protection, transplanting of individuals	Ex situ, in situ
Orchidaceae	*Epidendrum radioferens* (Ames, F.T.Hubb. & C.Schweinf.) Hágsater	RJS-3	Monjita colorada	Yes			0.002	BEA, BEM, Pal, Sol	Ixcatlán	Gathering, protection, transplanting of individuals	Ex situ, in situ
Orchidaceae	*Euchile karwinskii* (Mart.) Christenson	RJS-1	Monjita amarilla	Yes		Yes	0.002	BEA, Pal, Sol	Ixcatlán	Gathering, protection, propagation, transplanting of individuals	Ex situ, in situ
Orchidaceae	*Laelia albida* Bateman ex Lindl.	ERL-126	Monjita blanca	Yes			0.002	Pal, Sol, TS	Ixcatlán	Gathering, protection, propagation, transplanting of individuals	Ex situ, in situ
Orchidaceae	*Laelia anceps* Lindl.	SRL-1541	Monjita morada	Yes			0.002	AA, Pal, Sol	Ixcatlán	Gathering, protection, propagation, transplanting of individuals	Ex situ, in situ
Orchidaceae	*Rhynchostele maculata* (Lex.) Soto Arenas & Salazar	ERL-173, SRL-1476	Monjita pinta	Yes			0.002	BEA, BEM, Pal, Sol	Ixcatlán	Gathering, protection, transplanting of individuals	Ex situ, in situ
Orchidaceae	*Spiranthes* sp.	RLF-208	Monjita de peña	Yes			0	Iz	Ixcatlán	Gathering	In situ
Orobanchaceae	*Castilleja tenuifolia* M.Martens & Galeotti	SRL-117, SRL-223, SRL-329, SRL-1438, SRL-1485	Romero cimarrón	Yes		Yes	0	BEA, BN, Me, Palm	Ixcatlán	Forage, gathering	In situ
Orobanchaceae	*Conopholis alpina* Liebm.	SRL-218, SRL-1481	Flor de elote			Yes	0	BEA, Pal	Ixcatlán	Forage, gathering	In situ
Orobanchaceae	*Lamourouxia dasyantha* (Cham. & Schltdl.) W.R.Ernst	SRL-1379, SRL-1429	Lisión	Yes			0	BEA, BEC, BEM, Me	Ixcatlán	Gathering	In situ
Orobanchaceae	*Lamourouxia viscosa* Kunth	RLF-209, SRL-372, SRL-1292	Moco de pavo, flor de miel	Yes			0	Iz, Pal, Palm	Ixcatlán	Gathering, tolerance	In situ
Oxalidaceae	*Oxalis aff. latifolia* Kunth	ERL-75, RLF-142, SRL-148	Coyule		Yes		0	Iz, Me, Sol, TS	Ixcatlán	Forage, gathering, protection, tolerance	In situ
Oxalidaceae	*Oxalis aff. nelsonii* (Small) R.Knuth	SRL-1273	Coyule		Yes		0	Iz, Sol	Ixcatlán	Forage, gathering, protection, propagation	Ex situ, in situ
Oxalidaceae	*Oxalis* sp.	RLF-139	Coyule delgado		Yes		0	BEA, BEC, BN, Me	Ixcatlán	Forage, gathering	In situ
Papaveracea	*Argemone mexicana* L.	ERL-244, RLF-180, SRL-455	Chicalote	Yes		Yes	0	Bal, Pal, Sol, TS	Ixcatlán	Gathering, tolerance, uproot	In situ
Passifloraceae	*Passiflora bryonioides* Kunth	SRL-1148	Granadilla		Yes		0	Sol	Ixcatlán	Gathering, protection, tolerance	In situ
Passifloraceae	*Passiflora suberosa* L.	SRL-444, SRL-1164, SRL-1165				Yes	0	Sol	Ixcatlán	Gathering, tolerance	In situ
Passifloraceae	*Turnera diffusa* Willd. ex Schult.	SRL-1220, SRL-1356, SRL-1467	Tamorreal		Yes	Yes	0	CaCe, SB, Sol	Ixcatlán	Gathering, protection, transplanting of individuals	Ex situ, in situ
Phytolaccaceae	*Phytolacca icosandra* L.	RLF-236				Yes	0	Iz	Ixcatlán	Gathering	In situ
Piperaceae	*Peperomia quadrifolia* (L.) Kunth	ERL-146, SRL-1404, 1430	Verdolaga		Yes		0.014	BEM	Ixcatlán	Gathering, protection, transplanting of individuals	Ex situ, in situ
Plantaginaceae	*Bacopa monnieri* (L.) Wettst.	SRL-301, SRL-1132	Verdolaga de agua		Yes	Yes	0	Paz, VR	Ixcatlán	Forage, gathering	In situ
Plantaginaceae	*Penstemon barbatus* (Cav.) Roth	RLF-23, RLF-49, SRL-133, SRL-464, SRL-1314	Bandera	Yes		Yes	0	BEA, Palm	Ixcatlán	Gathering	In situ
Plantaginaceae	*Russelia obtusata* S.F.Blake	RLF-263, SRL-234, SRL-342, SRL-424, SRL-1494	Bandera			Yes	0	BEA, BG, BN, Me	Ixcatlán	Gathering	In situ
Plantaginaceae		SRL-1198	Bandera			Yes	0	BEA	Ixcatlán	Gathering	In situ
Poaceae	*Piptochaetium fimbriatum* (Humb., Bonpl. & Kunth) Hitchc.	RLF-137, SRL-260, SRL-413	Pasto			Yes	0.038	BEA, BG, Me, Paz	Ixcatlán	Forage, gathering	In situ
Poaceae	*Setaria grisebachii* E.Fourn.	RLF-231,RL-358	Pasto de semilla			Yes	0.038	Iz, Palm, Paz	Ixcatlán	Forage, gathering	In situ
Poaceae		SRL-311	Pasto de semilla			Yes	0.038	Paz	Ixcatlán	Forage, gathering	In situ
Polemoniaceae	*Loeselia caerulea* (Cav.) G.Don	RLF-265, SRL-96, SRL-353, SRL-1267, SRL-1282, SRL-1364, SRL-1401, SRL-1458				Yes	0	BEA, BN, CaCe, Me, Pal, Palm	Ixcatlán	Forage, gathering	In situ
Polygalaceae	*Polygala scoparia* Kunth	RLF-224, RLF-287				Yes	0	BN, Iz	Ixcatlán	Forage, gathering	In situ
Portulacaceae	Portulaca oleracea L.	Photo record	Verdolaga de suelo		Yes		0	Bal, Sol, TS	Ixcatlán	Enhancement, gathering, tolerance, transplanting of individuals, uproot	In situ
Primulaceae	*Anagallis arvensis* L.	ERL-108, ERL-228, RLF-200, SRL-87, SRL-100, SRL-1133	Jabonera, hierba de pollo		Yes	Yes	0	Bal, BN, Iz, Palm, Sol, TS	Naturalized, from other continents	Gathering, tolerance	Ex situ
Pteridaceae	*Adiantum poiretii* Wikstr.	SRL-202,SRL-427				Yes	0	BG, VR	Ixcatlán	Gathering	In situ
Ranunculaceae	*Delphinium bicornutum* Hemsl.	SRL-1200	Conejito	Yes			0	BEA	Ixcatlán	Gathering	In situ
Ranunculaceae	*Thalictrum gibbosum* Lecoy.	RLF-212, RLF-302	Chichicasle			Yes	0	Iz	Ixcatlán	Gathering	In situ
Rhamnaceae	*Condalia mexicana* Schltdl.	RLF-86, SRL-457, SRL-1147	Espino capulín		Yes		0	Pal, Sol	Ixcatlán	Gathering, tolerance	In situ
Rosaceae	*Crataegus mexicana* Moc. & Sess‚ ex DC	SRL-1424	Tejocote		Yes		0.002	Paz, TS	Ixcatlán	Gathering, propagation, tolerance	In situ
Rosaceae	*Lindleya mespiloides* Kunth	SRL-1223, SRL-1493	Hierba de pajarito, campanita grande	Yes		Yes	0	Me, SB	Ixcatlán	Gathering	In situ
Rosaceae	*Malacomeles denticulata* (Kunth) G.N.Jones	RLF-10, RLF-243, SRL-261, SRL-338, SRL-474, SRL-1257, SRL-1258	Tlasisle		Yes	Yes	0	BEA, BEC, BN, Iz, Me, Palm, TS	Ixcatlán	Forage, gathering, tolerance	In situ
Rubiaceae	*Bouvardia longiflora* (Cav.) Kunth	Photo record	Huele de noche	Yes			0	Me	Ixcatlán	Gathering	In situ
Rubiaceae	*Bouvardia ternifolia* (Cav.) Schltdl.	RLF-41, RLF-166, SRL-262, SRL-334, SRL-1417	Ventorilla, flor de triste	Yes		Yes	0	BEA, BEC, Me, Palm, Paz, TS	Ixcatlán	Forage, gathering, tolerance, uproot	In situ
Rubiaceae	*Chiococca alba* (L.) Hitchc.	SRL-336, SRL-470, SRL-1111, SRL-1331, SRL-1441	Campanita	Yes			0.002	CaCe, Me, Sol	Ixcatlán	Gathering, protection, transplanting of individuals	Ex situ, in situ
Rubiaceae	*Crusea* sp.	RLF-136, SRL-1180		Yes			0	Me, Palm	Ixcatlán	Gathering	In situ
Rubiaceae	*Galium* sp.	RLF-82, RLF-280, SRL-344				Yes	0	BEA, Me, Pal, Palm	Ixcatlán	Gathering	In situ
Rubiaceae	*Randia capitata* DC.	RLF-281, SRL-1208	Limoncito de coyote			Yes	0	BEA, Pal, VR	Ixcatlán	Gathering	In situ
Rutaceae	*Ptelea trifoliata* L.	ERL-196, RLF-27, RLF-308, SRL-274, SRL-466, SRL-467	Hierba de zorrillo			Yes	0.002	BEA, BEC, BG, BN, Iz, Me, Palm, Sol, TS	Ixcatlán	Gathering, tolerance	In situ
Rutaceae	*Zanthoxylum* sp.	SRL-1348				Yes	0	CaCe	Ixcatlán	Gathering	In situ
Santalaceae	*Phoradendron* sp.	RLF-228, SRL-1268	Injerto			Yes	0	Iz, Me	Ixcatlán	Gathering, uproot	In situ
Selaginellaceae	*Selaginella lepidophylla* (Hook. & Grev.) Spring	SRL-374, SRL-1497				Yes	0	BEA, Me	Ixcatlán	Gathering	In situ
Solanaceae	*Capsicum annuum* L.	ERL-165, ERL-204	Chilar de monte		Yes		0.006	SB, Sol	Ixcatlán	Gathering	Ex situ, in situ
Solanaceae	*Capsicum* sp.	RLF-135				Yes	0	Me	Ixcatlán	Gathering	In situ
Solanaceae	*Jaltomata procumbens* (Cav.) J.L.Gentry	SRL-180, SRL-1297	Hierba mora		Yes	Yes	0	Palm, Sol	Ixcatlán	Gathering, tolerance	In situ
Solanaceae	*Lycianthes ciliolata* (M.Martens & Galeotti) Bitter	SRL-1149	Ojo de toro		Yes	Yes	0	BEA, BG, Pal, Palm, Sol	Ixcatlán	Gathering, tolerance	In situ
Solanaceae	*Nicotiana glauca* Graham	ERL-37, RLF-105, SRL-171, SRL-1274	Gigante			Yes	0	Bal, Pal, Sol, TS	Naturalized, from other parts of American Continent	Gathering, tolerance	Ex situ
Solanaceae	*Physalis philadelphica* Lam.	ERL-36, ERL-63, ERL-64, ERL-113, RLF-312, SRL-26, SRL-1138, SRL-1298	Miltomate, tomate, tomate de milpa		Yes	Yes	0	Sol, Ts	Ixcatlán	Gathering, enhancement, protection, propagation, tolerance, transplanting of individuals	In situ
Solanaceae	*Solanum americanum* Mill.	SRL-1234	Ticungo		Yes		0	Sol	Ixcatlán	Gathering, tolerance	In situ
Solanaceae	*Solanum erianthum* D.Don.	ERL-91	Tepozán			Yes	0	Sol	Ixcatlán	Gathering, tolerance	In situ
Solanaceae	*Solanum lanceolatum* Cav	ERL-195	Tepozán			Yes	0	BEA, BEC, BG, Palm, Sol	Ixcatlán	Gathering, tolerance	In situ
Solanaceae	*Solanum lesteri* Hawkes & Hjert.	RLF-151	Hierba del tomate pinto		Yes		0	Paz	Ixcatlán	Gathering	In situ
Solanaceae	*Solanum rostratum* Dunal	SRL-380	Chicalote de burro			Yes	0	BEA	Ixcatlán	Gathering	In situ
Solanaceae	*Solanum rudepannum* Dunal	RLF-22, RLF-95, RLF-120, RLF-275, SRL-128, SRL-302	Tepozán		Yes	Yes	0	Sol, BEA, BEC, Me, Pal, Paz	Ixcatlán	Gathering	In situ
Thelypteridaceae	*Thelypteris* sp.	SRL-161, RLF-303		Yes			0	BEA, Iz, Pal	Ixcatlán	Gathering	In situ
Tropaeolaceae	*Tropaeolum majus* L.	ERL-18, ERL-89, RLF-182, SRL-60, SRL-196	Mastuerzo	Yes		Yes	0	Sol	Naturalized, from other parts of American Continent	Gathering, enhancement, protection, propagation, tolerance	Ex situ
Urticaceae	*Parietaria pensylvanica* Muhl. ex Willd.	ERL-73, RLF-88, RLF-266, SRL-18	Paletaria			Yes	0	BEA, Pal, Sol, VR	Ixcatlán	Gathering, tolerance	In situ
Urticaceae	*Urera caracasana* (Jacq.) Gaudich. ex Griseb.	SRL-1543	Chichicasle			Yes	0	Sol	Ixcatlán	Gathering, tolerance	In situ
Verbenaceae	*Glandularia elegans* (Kunth) Umber	RLF-5, SRL-110, SRL-279, SRL-1326, SRL-1479				Yes	0	Bal, BEA, BN, Sol	Ixcatlán	Gathering	In situ
Verbenaceae	*Lantana achyranthifolia* Desf.	RLF-61, RLF-62, SRL-109, SRL-152, SRL-369, SRL-1296	Hierba buena de monte			Yes	0	BEA, BN, Me, Pal, Palm	Ixcatlán	Forage, gathering, tolerance, uproot	In situ
Verbenaceae	*Lantana camara* L.	RLF-197, SRL-115, SRL-459, SRL-1112, SRL-1154, SRL-1169, SRL-1365	Tiundica, siete negritos		Yes	Yes	0	BEA, BEC, BN, CaCe, Iz, Me, Palm, Sol	Ixcatlán	Forage, gathering, protection, transplanting of individuals	Ex situ, in situ
Verbenaceae	*Lantana velutina* M.Martens & Galeotti	ERL-185, RLF-31, RLF-204, SRL-272, SRL-1115, SRL-1168	Tiundica blanca, cinco negritos		Yes	Yes	0	BEA, BEC, BN, Iz, Me, Pal, Palm, Sol	Ixcatlán	Gathering, tolerance, transplanting of individuals	Ex situ, in situ
Verbenaceae	*Lippia graveolens* Kunth		Oreganillo, salvarreal de castilla		Yes	Yes	0	CaCe, Me, Pal	Ixcatlán	Forage, gathering	In situ
Verbenaceae	*Lippia oaxacana* B.L.Rob. & Greenm.	SRL-71, SRL-1378, SRL-1454, SRL-1549	Salvarreal		Yes	Yes	0.014	Me, Sol	Ixcatlán	Gathering, protection, transplanting of individuals	Ex situ, in situ
Vitaceae	*Cissus* sp.	RLF-101, RLF-173, SRL-1373, SRL-1535	Tripa de diablo			Yes	0	CaCe, Sol, TS	Ixcatlán	Gathering, tolerance, uproot	In situ
			Octavillo	Yes			0	BEM	Ixcatlán	Gathering	In situ

^a^Key to collector. ERL = Erandi Rivera Lozoya; RJS = José Rosario Jiménez Salazar; SRL = Selene Rangel Landa; RLF = Ricardo Lemus Fernández

^b^Key to vegetation type. AA = ancient settlements; Bal = urban secondary vegetation; BEA = *Quercus liebmanni* and *Q. laeta forest*; BEC = *Quercus urbanni* forest; BEM = *Quercus* spp.forest; BG = gallery forest (*Taxodium huegelii*); BN = *Juniperus flaccida* forest; CaCe = *Cephalocereus colummna-trajanni* shrubland; CaMy = *Pseudomytrocereus fulviceps* shrubland; Iz = Izotal (shrubland dominated by rosettes); Me = Mexical; Pal = mescal factories; Palm = palm shrubland of *Brahea dulcis*; Paz = grassland; SB = tropical dry forest; Sol = homegardens; TS = agricultural fields; VR = riparian vegetation

**Table 5:** Sociocultural parameters estimated for species considered in in-depth studies

ID	Species	Us	SIB	SIU	Con	UF	Var	EI	RI	SCS	UP^a^	HEf^a^	HTo^a^
Ceremonial													
Bbif	*Bursera biflora* (Rose) Standl.	7	0	0.028	1	5	1	1, 3	1, 2, 3	1, 2, 3, 6	4	3	9
Blon	*Bouvardia longiflora* (Cav.) Kunth	1	0	0.006	0.01	3	0	1	1	0	2	1	1
Bstr	*Beaucarnea stricta* Lem.	2	0.005	0	1	4	0	1	1, 2, 3	1	1	2	4
Calb	*Chiococca alba* (L.) Hitchc.	3	0.002	0.066	0.99	4	0	1	1, 2	0	2	1	0
Dser	*Dasylirion serratifolium* (Karw. ex Schult. & Schult.f.) Zucc.	5	0.010	0	1	4	2	1	1, 2, 3	1	2	3	7
Dspp	*Dahlia apiculata* (Sherff) P.D.Sorensen; *Dahlia coccinea* Cav.	2	0	0	0.12	4	1	1	1	0	2	1	1
Ekar	*Euchile karwinskii* (Mart.) Christenson	3	0.002	0.033	0.99	3.5	0	1	1,3	0	2	3	5
Erad	*Epidendrum radioferens* (Ames, F.T.Hubb. & C.Schweinf.) Hágsater	2	0.002	0	0.85	3	0	1	1,3	0	3	1	1
Lalb	*Laelia albida* Bateman ex Lindl.	2	0.002	0.052	0.77	4	0	1	1,3	0	2	1	2
Ldas	*Lamourouxia dasyantha* (Cham. & Schltdl.) W.R.Ernst	2	0	0.039	0.17	3	2	1	1, 2	0, 2	2	1	3
Lgla	*Litsea glaucescens* Kunth	3	0.010	0	1	6	0	1	1, 2, 3	3, 4	1	3	3
Lmes	*Lindleya mespiloides* Kunth	2	0	0.015	0.05	4	0	1	1, 2	0, 2	2	1	1
Mdep	*Meteorium deppei* (Hornsch. ex Müll. Hal.) Mitt.	2	0	0	1	4	1	1	3	0, 2	1	2	6
Octa	“Octavillo”	2	0	0.025	0.01	2	0	1	1, 2	0, 1, 2	1	2	3
Prub	*Plumeria rubra* L.	2	0	0.007	0.05	2	2	1, 3	1	0	2	3	3
Rmac	*Rhynchostele maculate* (Lex.) Soto Arenas & Salazar	2	0.002	0.005	0.92	2	0	1	1,3	1	2	3	5
Spur	*Salvia purpurea* Cav.	3	0	0.016	0.05	3.3	2	1	1	0	2	1	1
Tgra	*Tillandsia grandis* Schltdl.	2	0	0.009	1	5	0	1	3	1	1	2	9
Tluc	*Tagetes lucida* Cav.	4	0.003	0.007	0.5	1	0	1	1, 2	0, 3	2	1	1
Tusn	*Tillandsia usneoides* (L.) L.	5	0	0	1	3	0	1	1	0, 2	1	1	1
Edible													
Acris	*Anoda cristata* (L.) Schltdl.	4	0	0.012	0.05	2	0	1	1	0	3	2	1
Ahyb	*Amaranthus hybridus* L.	3	0.024	0.252	0.95	4.5	3	1, 3	1, 2	4	1	3	1
Aker	*Agave kerchovei* Lem.	4	0	0.015	0.2	3	0	1	1	0, 1	3	3	4
Apot	*Agave potatorum* Zucc.	8	0.057	0.072	0.25	3.5	2	1, 2, 3, 4	1, 2, 3	1, 4, 6	5	3	7
Bdul	*Brahea dulcis* (Kunth) Mart.	11	0.106	0.002	1	2	0	1, 2, 3, 4	1,3	1, 3, 6	5	3	7
Cber	*Chenopodium berlandieri* Moq.	2	0.006	0.022	0.15	3	3	1	1, 2	0	1	3	1
Crme	*Crataegus mexicana* Moc. & Sess‚ ex DC	1	0.002	0.011	0.35	4	3	1, 2, 3	1, 2	0, 1, 2, 3, 6	1	2	1
Damb	*Dysphania ambrosioides* (L.) Mosyakin & Clemants	3	0.065	0.024	1	6	3	1, 3	1, 2	3, 4, 6	2	2	1
Dser	*Dasylirion serratifolium* (Karw. ex Schult. & Schult.f.) Zucc.	5	0.010	0.110	0.95	4	2	1	1, 2, 3	0, 1	2	3	7
Lgla	*Litsea glaucescens* Kunth	3	0.010	0.026	0.14	3.5	0	1	1, 2, 3	3, 4	1	3	3
Lspp	*Lantana camara* L.; *L*. *velutina* M.Martens & Galeotti	5	0	0	0.05	4.5	1	1	1	0	2	1	1
Mspp	*Mammillaria haageana* Pfeiff.; *Mammillaria sphacelata* Mart.	3	0	0	0.05	3.3	1	1	1, 2	0	2	1	1
Noff	*Nasturtium officinale* R.Br.	1	0.002	0.013	0.15	2.5	0	1	1	0	1	1	1
Olas	*Opuntia lasiacantha* Pfeiff.	6	0.042	0.043	1	6	3	1, 3	1, 2	1, 2, 4, 6	5	3	5
Ospp	*Oxalis* aff. *latifolia* Kunth; *Oxalis* aff. *nelsonii* (Small) R.Knuth	2	0	0.007	0.45	3	1	1, 3	1, 2	0	1	3	1
Plin	*Porophyllum linaria* (Cav.) DC.	2	0	0.078	0.95	6	0	1, 3	1, 2	4, 6	3	1	1
Pole	*Portulaca oleracea* L.	3	0	0.010	0.05	4	0	1	1	0	1	3	1
Pphi	*Physalis philadelphica* Lam.	2	0	0.015	1	6	3	1, 2, 3	1,3	2, 3, 5, 6	1	3	1
Pqua	*Peperomia quadrifolia* (L.) Kunth	2	0.014	0.070	0.95	4	3	1	1, 2	0	1	3	1
Prud	*Porophyllum ruderale subsp*. *macrocephalum* (DC.) R.R.Johnson	1	0.004	0.161	0.9	5	0	1, 3	1, 2	0	1	1	1
Medicinal													
Amai	*Ageratina mairetiana* (DC.) R.M.King & H.Rob.	3	0.014	0.150	0.85	2	2	1	1, 2	1, 3, 4	3	3	1
Apsi	*Ambrosia psilostachya* DC.	1	0	0.032	0.85	2.5	0	1	1, 2	1, 2, 3, 4	1	3	1
Bsal	*Barkleyanthus salicifolius* (Kunth) H.Rob. & Brettell	6	0.003	0.029	0.85	3	0	1	1, 2	4	3	2	3
Clme	*Clinopodium mexicanum* (Benth.) Govaerts	2	0	0.136	0.85	4	0	1	1, 2	1, 3, 4	2	3	1
Cmex	*Chrysactinia mexicana* A.Gray	1	0	0.017	0.85	3.5	0	1	1, 2	1, 3, 4	3	3	1
Dcar	*Dalea carthagenensis* (Jacq.) J.F.Macbr.	2	0	0.010	0.85	2.5	0	1	1	0	1	3	1
Gglu	*Gymnosperma glutinosum* (Spreng.) Less.	3	0.002	0.031	0.85	0.5	0	1	1	0	1	2	0
Ginu	*Grindelia inuloides* Willd.	1	0.029	0.094	0.85	2.5	0	1	1, 2	1, 2, 3, 4	1	3	1
Loax	*Lippia oaxacana* B.L.Rob. & Greenm.	2	0.014	0.264	0.85	3.5	0	1	1, 2	1, 3, 4	1	3	1
Mpar	*Malva parviflora* L.	3	0	0.032	0.85	3.5	0	1	1, 2	0	3	2	1
Mpur	*Matelea purpusii* Woodson	2	0	0.015	0.85	3.5	0	1	1, 2	0	2	3	1
Mvul	*Marrubium vulgare* L.	1	0	0.056	0.85	3	0	1	1, 2	0	1	2	1
Ppen	*Parietaria pensylvanica* Muhl. ex Willd.	1	0	0.016	0.85	1	0	1	1, 2	0, 2	1	2	1
Pros	*Pinaropappus roseus* (Less.) Less.	1	0	0.012	0.85	1	0	1	1	0	3	1	1
Rcom	*Ricinus communis* L.	4	0	0.016	0.85	3	3	1	1, 2	0	2	2	1
Spra	*Senecio praecox* (Cav.) DC.	3	0	0.009	0.85	1	0	1	1, 2	3, 4	2	3	1
Tdif	*Turnera diffusa* Willd. ex Schult.	2	0	0.037	0.85	2	0	1	1,2	3	1	1	1
Tluc	*Tagetes lucida* Cav.	4	0.003	0.052	0.85	4.5	0	1	1, 2	0, 3	2	1	1
Apot	*Agave potatorum* Zucc.^a^	8	0.057	0.039	0.85	2	2	1, 2, 3, 4	1, 2, 3	1, 4, 6	5	3	7
Qacu	*Quercus acutifolia* Née^a^	7	0.072	0.010	0.85	1	1	1, 3	1,3	0, 2	4	2	11

*ID* identification tag assigned to the species analyzed, *Us* uses number, *SIB* Sutrop index for plants considered basic to life, *SIU* Sutrop index by use type, *Con* consumption, *UF* use frequency, *Var* recognized variants, *EI* economic interchange, *RI* reciprocity interchange, *SCS* sociocultural strategies, *UP* useful parts, *HEf* harvest effort, *HTo* tools used for harvest

^a^Excluded variables and species in the performance of principal component analyses (PCA) and canonical correspondence analyses

Commercialization of ceremonial wild plants is uncommon, except the resin of *Bursera* spp., which is used for celebrating the day of the dead. People used to share part of flowers collected in forests or managed in homegardens (mainly *Chiococca alba*, *Lindleya mespiloides*, orchids, and copal resin (*Bursera* spp.)) and give them as presents to people who organize the communitarian feasts. *Dasylirion serratifolium*, *Beaucarnea stricta*, and *Tillandsia grandis* are involved in practices of reciprocity among most of the local households in communitarian feasts (Table 5).

Ornamenting of altars is mostly attended with plants cultivated in homegardens. Due to the scarcity of copal and other plants used in ceremonies, people practice gathering them in different sites throughout their territory (Tables 5 and 6). In addition, we recorded storing of copal resin for use throughout the year (Table 5).

**Table 6:** Meaningful consultant’s commentaries about the use, abundance, and their motives to manage plants

Use	ID	Species	Management motives and observations about use and availability
Ceremonial	Bbif	*Bursera biflora* (Rose) Standl.	Trees are abundant, but copal could becomes scarce. Care should be taken to not damage the tree, to tree continue producing the copal. Only the one produced naturally, by the worm [butterfly larvae] in hot terrain is good for burning. Not [transplant or cultivation] because the tree would not survive or produce copal here in the village. I have a little tree that I take out of the forest for the luxury of my house and I hope that someday it will produce copal, although maybe it would not be enough or good. I plant a stick, there in the mountain where I go to collect the “copal”, I did to see if it [roots].
Ceremonial	Blon	*Bouvardia longiflora* (Cav.) Kunth	Now it is almost no longer used, there are other flowers [flowers of introduced species].
Ceremonial	Bstr	*Beaucarnea stricta* Lem.	The gathering is dangerous, the plant is in very difficult places to walk. Care must be taken to not injure the tree, the [apical meristem], so that the plant continues to produce, sometimes the tree is damaged, but that should not be done.
Ceremonial	Calb	*Chiococca alba* (L.) Hitchc.	Before it was used [to offer it] in the church, but now no longer because they criticize, only is placed on the altars of the houses. I really like its flowers, its scent, I put it on my altar. Once I brought a little tree to the house but It do not survive. Out of curiosity I try to [cultivate], but it does not [germinate].
Ceremonial	Dser	*Dasylirion serratifolium* (Karw. ex Schult. & Schult.f.) Zucc.	It should leave part of the trunk, if there is good rain it can sprout. It has not occurred to us to bring the plant to the village, “it is natural” [it occurs naturally in the field], we always have found it to make the adornments.
Ceremonial	Dspp	*Dahlia apiculata* (Sherff) P.D.Sorensen; *Dahlia coccinea* Cav.	I like to have them in the house, for luxury [ornamental use] and put the flowers on the altar.
Ceremonial	Ekar	*Euchile karwinskii* (Mart.) Christenson	It must remain [peudobulbs] to have it for another time, they are the ornament of the trees [in the forest]. After the flower dries, the (pseudobulb] is placed in some tree in the house, and so it is going to have for luxury [ornamental use] and have flowers to adornment the altar. When I am gathering firewood and I cut a branch that have “monjitas” [orchids], sometimes I transplant it in other branch and sometimes I bring it to the house.
Ceremonial	Erad	*Epidendrum radioferens* (Ames, F.T.Hubb. & C.Schweinf.) Hágsater	It must remain [peudobulbs] to have it for another time. They are the ornament of the trees [in the forest]. After the flower dries, is placed in some tree in the house, and so it is going to have for luxury [ornamental use] and have flowers to adornment the altar, however it is difficult, it is a delicate plant.
Ceremonial	Lalb	*Laelia albida* Bateman ex Lindl.	I take care it [cultivation] to have flowers for the altar in Todos Santos [celebration] and for the luxury of my home.
Ceremonial	Ldas	*Lamourouxia dasyantha* (Cham. & Schltdl.) W.R.Ernst	There is much when rain is good, but when it is not given, I use whatever available flower.
Ceremonial	Lgla	*Litsea glaucescens* Kunth	There is a lot in the forest, there is always when it is needed and the tree will regrow if you do not hurt it. I have not had the curiosity [transplanting] and the need because there is [enough], and there is also little terrain to have it maybe it will dry. Out of curiosity, I put some seeds but they did not germinate.
Ceremonial	Lmes	*Lindleya mespiloides* Kunth	There is a lot in the forest, but sometimes there are no flowers due to the drought.
Ceremonial	Mdep	*Meteorium deppei* (Hornsch. ex Müll. Hal.) Mitt.	After the celebration, I put it in my yard for luxury, but it dried.
Ceremonial	Octa	“Octavillo”	I have always found when I am going to collect, but sometimes, in order to not go up to the mountain, I better buy others [other plants in regional markets]. I think it would not survive [transplanting, cultivation], is a delicate plant and its environment is very different, more template.
Ceremonial	Prub	*Plumeria rubra* L.	I have not tried [propagation], I have not had the curiosity, I like it a lot but I do not try to have it, but there are people that have it. I plant a stick to have the tree here in the house, but it rotted, maybe I try again later.
Ceremonial	Rmac	*Rhynchostele maculate* (Lex.) Soto Arenas & Salazar	It must remain [peudobulbs] to have it for another time. They are the ornament of the trees [in the forest]. After the flower dries, the [pseudobulb] is placed in some tree in the house, and so it is going to have for luxury [ornamental use] and have flowers to adornment the altar. It is difficult take care of it because it is delicate, but it is a pride to have it.
Ceremonial	Spur	*Salvia purpurea* Cav.	Used more before. There is much when rain is good, but when it is not given, I use whatever available flower, now there are other flowers [introduced that are grown or bought in local stores]. Once I take one from the mountain, to have the flowers for my altar and luxury of the house, but it dried and I have not tried again.
Ceremonial	Tgra	*Tillandsia grandis* Schltdl.	There has always been when it is needed. Once I brought some small plants [transplanting] but dried, is very delicate, needs its natural environment.
Ceremonial	Tluc	*Tagetes lucida* Cav.	There is much when rain is good, but when it is not given, I use whatever available flower.
Ceremonial	Tusn	*Tillandsia usneoides* (L.) L.	I have this plant, I bring it from the mountain and from the adornment of holidays, it is for decoration of my trees and also to feed the cattle when there is nothing, to clean the frets, for what is could needed here I got it near, in my house.
Edible	Acris	*Anoda cristata* (L.) Schltdl.	Before the people collected it, they gathered. Now it is scarce and people say that who eats it does not have money to buy food.
Edible	Ahyb	*Amaranthus hybridus* L.	It is very tasty, it is important to eat it, but it is left to the time and the rain, there has been no need to cultivate it, it is only left on the edge of the cropland to produce seed. There are different colors but if it is “tierno” [shoots] taste does not change, but others prefer the green. When there are a lot and is “sazón” [mature] it could damage the other plants so it is plucked.
Edible	Aker	*Agave kerchovei* Lem.	People say that when someone eats “cacayas” [floral buttons] it’s because they do not have money for food, but we like it. Only is gathered, it is close, it is not necessary to propagate it.
Edible	Apot	*Agave potatorum* Zucc.	This “cacaya” was eaten a lot, was eaten boiled with sauce when there was nothing else or when corn was scarce it was mixed with the nixtamal [boiled corn] to raise it to make the tortillas. When we cooked maguey with coyule [Oxalis spp.] we gave to friends and relatives and other part is for sell it. Now people have it in their fields for mescal, but it was getting scarce, now they are sowing it [mescal producers and external institutions]. Its leaf and thorns vary in shape and color, its size is different, ones gives more mescal, although we like it to be large we cut everything. When we collect seed for [cultivate] it, we go to sites where we know the maguey is big and produce more mescal, others only get the first [capsules with seeds] available.
Edible	Bdul	*Brahea dulcis* (Kunth) Mart.	When a field is opened [for agriculture], the palm is left, it is our sustenance, the hat. I do not wave the hat but my neighbors do it, is the sustenance of the town, it is the motive because I left it in my terrain [tolerance].
Edible	Cber	*Chenopodium berlandieri* Moq.	Abundance: Before there was more because they no longer work the land as the older. On the edge of the land some are left (tolerated) to produce seeds and there are for the next year.
Edible	Crme	*Crataegus mexicana* Moc. & Sess‚ ex DC	Before there were more, now no one cares for them, the animals eat [cattle]. There are with large and small fruit, with sweet and sour taste. I tried to [propagates] but it does not [germinate].
Edible	Damb	*Dysphania ambrosioides* (L.) Mosyakin & Clemants	Sometimes my neighbor and my aunt ask me for some of it and as I have, I give them a little. I saw a little plant that I liked for its large and green leaves and brought it to my house, I take care of it and now I have all the time. There are green, purple and “criollo” (from the store), the last does not have smell, nor taste.
Edible	Dser	*Dasylirion serratifolium* (*Karw. ex Schult. & Schult.f.*) *Zucc.*	Although the plant is abundant, the “manita” [Floral buttons ] becomes scarce because of the drought, when that happens we were left with the desire to eat it that year. It has not occurred to me to take the “manita” plant to the village, “it is natural”. There are green “manitas” that are sweet and purples that are bitter, but at the whim we eat the same two.
Edible	Lgla	*Litsea glaucescens* Kunth	For food it requires little, a few twigs. I have not had the curiosity, the need [propagation], I only go to the field and collect it. Out of curiosity, I put some seeds but they did not germinate.
Edible	Lspp	*Lantana camara* L.; *L. velutina* M.Martens & Galeotti	They eat it when they go to the field, but they are not sweet, they are simple.
Edible	Mspp	*Mammillaria haageana* Pfeiff.; *Mammillaria sphacelata* Mart.	I brought one to my house for luxury, not to eat the “chilitos” [fruits], I like the way it looks.
Edible	Noff	*Nasturtium officinale* R.Br.	It is no longer consumed because there is no one who collects it. When harvested, the root must be left to it could sprout.
Edible	Olas	*Opuntia lasiacantha* Pfeiff.	As I have many plants I always have, I give it to my family when they ask me and sometimes other people come to ask me, sometimes I give them and others I sell it depending on how much it is. I brought a “penquita” [cladode] and now all those who fall and take root I care of them because it is the “nopal” that I like, those that come from outside are not good. There are some more spiny than others and they give “tunas” [fruits] of different color.
Edible	Ospp	*Oxalis* aff. *latifolia* Kunth; *Oxalis* aff. *nelsonii (*Small) R.Knuth)	I brought this [*Oxali*s plant] out of curiosity, for luxury of the house [ornamental], when we want make the “conserva” [Traditional dish prepared with *Agave potatorum* stems and *Oxalis* leaves] we go to the mountain where it grow big.
Edible	Plin	*Porophyllum linaria* (Cav.) DC.	Its left on the edge of the cropland to produce seed. Some people have had the curiosity to cultivate it, they have it all the time, sometimes they give me a little. I only like the plants that I have inside of the “corralito” [space inside the yard delimited by a fence] or that are in crop lands, it is abundant in roads and the yard but is nasty by the animals.
Edible	Pole	*Portulaca oleracea* L.	I leave some plants to flower and give seed to have in abundance next year, although when it is a lot it is plucked.
Edible	Pphi	*Physalis philadelphica* Lam.	Last year was good [good production], it reach to give 6 kilos to my brothers who live outside It is abundant, but it is because we take care of it. I leave some [fruits] so that the next year can continue, in homegardens is watered, fertilized, so that they can produce [fruits]. There is “milomate” of the maize crop land, “dulce” (sweet) from the wheat cropland and one big that we get in the store, the last one is not so good and to have [manage and cultivate] we choose the miltomate and the sweet, of which it is pretty [big].
Edible	Pqua	*Peperomia quadrifolia* (L.) Kunth	Now that we are old and we can not go to the mountain, we just eat it when my son-in-law shares us. There is, but it is retired, in rains it is more [juicy]. The one from Gandudo is more tastier. Somebody brought to have here, but it dries, here is not their environment.
Edible	Prud	*Porophyllum ruderale* subsp. *macrocephalum* (DC.) R.R.Johnson	I have not had the curiosity to sow it, but there are some people who have it in their homes, they take care of it and have to eat all the year. When it is collected only the tender twigs should be cut so that it continues to sprout.
Medicinal	Amai	*Ageratina mairetiana* (DC.) R.M.King & H.Rob.	Only the twigs are cut, the rest is left and thus sprouts. Only the twigs are cut, if everything is harvested, it runs out. When there is one in the house or the agricultural field, is [tolerated].
Medicinal	Apsi	*Ambrosia psilostachya* DC.	In the harvest, the twigs are cut and the stem is left so it can sprout. We do not bring it to the house because we do not know if it will survive, we do not know what it need to produce. We store it because there is no in dry season.
Medicinal	Bsal	*Barkleyanthus salicifolius* (Kunth) H.Rob. & Brettell	Before, many people had it in their homes, now they do not like it so much. It is easy to have it, does not need care as fertilizer or irrigation. I have it, because when it is needed, I only go to the yard, besides it’s luxury [ornamental] for my house.
Medicinal	Clme	*Clinopodium mexicanum* (*Benth.*) *Govaerts*	It is not necessary to cut all the plant, only the twigs, leaving the stem can sprout and continues [be available]. I always have dry, it is more to drink, because it is almost not used as medicine. For medicine, it is collected when it’s needed, is not necessary to bring it [to the houses]. Here [mescal factory] it is natural [natural distribution], we only take care of it when is harvested and that the animals (cattle) do not foraged it.
Medicinal	Cmex	*Chrysactinia mexicana* A.Gray	I have not tried to bring it to the house, but if I would do it, it could be, to do not have to go by it, although I do not know if it could survive. I always have some of this plant, I let it dry and I keep it for when it is needed, when I go to the field and there are, I gather it, so I always have. When I need it and I do not have it stored, I ask someone to give me a little.
Medicinal	Dcar	*Dalea carthagenensis* (Jacq.) J.F.Macbr.	This is no longer used so much, but when I need it I’m going to gather it to the edge of town or somebody brings it to me
Medicinal	Gglu	*Gymnosperma glutinosum* (Spreng.) Less.	There is much everywhere, you only have to gather it when do you need it.
Medicinal	Ginu	*Grindelia inuloides* Willd.	There are those who have it (managed in the houses or dry), when it is needed, we asks them for it or we are going to look for it to field. I think it is not difficult, but maybe the soil did not help to survival of the one that I tries to propagate.
Medicinal	Loax	*Lippia oaxacana* B.L.Rob. & Greenm.	Only the twigs are cut so that it can sprout. When drying this plant does not lose its quality, it is very strong. We store it so we can have it when we need it. I worry that there is not [available when its needed], but I do not bring seedlings to the house because if I bring them and they dry, I will only run out them. I brought a little plant but it dried.
Medicinal	Mpar	*Malva parviflora* L.	I leave some plants on the edge to have it, but when there is a lot, it must be rooted out.
Medicinal	Mpur	*Matelea purpusii* Woodson	I brought it to my house because I’m [need it], so I always have it here.
Medicinal	Mvul	*Marrubium vulgare* L.	Is very resistant, while more you cut, more there are. I leave some plants on the edge to have it, but when there is a lot, it must be rooted out.
Medicinal	Ppen	*Parietaria pensylvanica* Muhl. ex Willd.	There are at the edge of the village, in my house I leave them in case that someday I would need it.
Medicinal	Pros	*Pinaropappus roseus* (Less.) Less.	Before it was used when it was at hand, there are others that are used for [the same].
Medicinal	Rcom	*Ricinus communis* L.	I have of the two [green and red] each one has its use, they are also luxury [ornamental]. I brought the first from the road, I transplant the [seedlings] and take care of them, there are those who have taken the seed of those that they need to sow it. When they are abundant, they have to be rooted out.
Medicinal	Spra	*Senecio praecox* (Cav.) DC.	Now little is used, before it was stored, now only a few use it. I brought a little plant, out of curiosity, now it is luxury of my house [ornamental] and by the time I need it I have it at hand.
Medicinal	Tdif	*Turnera diffusa* Willd. ex Schult.	When drying this plant does not lose its quality, it is very strong. When I need it and I do not have it stored, I ask someone to give me a little.
Medicinal	Tluc	*Tagetes lucida* Cav.	I always have dry for when it is needed, this plant does not lose its quality when is drying, it is very strong. If part of the stem is left it can sprout, it must be left to continue [be abundant].
Medicinal	Apot^a^	*Agave potatorum* Zucc.	Now few cooked the “conserva” [maguey stem cooked with *Oxalis* spp. leaves], but when they do it, they share it with their friends or they also sell it. The cacayas are eaten [flowers] when we meet one while walking in the field, to make mescal we have to go especially to cut the maguey and sometimes we have to buy it to other communities. Now there is scarce before there was here on the shore, now we have to walk to find, about three hours or more to [prepare] the mescal. Now [governmental] programs bring the maguey, we plant them in the fields and some [mescal producers] are already producing the plant, but it still lacks [time] to have it again.
Medicinal	Qacu^a^	*Quercus acutifolia* Née	When needed [for medicinal use] we look for it, just a few branches with tender leaves. It is also widely used by firewood. For wood, trees should not cut only the branches. I have two little trees, I brought acorns to feed my animals, but I leave some because I like these plants, but it is difficult they are delicate they hardly [germinate or survive], it takes a lot of patience and a lot of cares.

^a^Excluded variables and species in the performance of principal component analyses (PCA) and canonical correspondence analyses

Several species considered scarce in the wild are, however, enough for satisfying the needs of the community; this is particularly the case of *Tillandsia grandis* (Table 5). The availability of useful plants depends on seasonality, annual rainfall, and incidence of pests (Tables 5 and 6).

Gathering was the only practice for 28 ornamental species (Table 3); species used for ornamenting the altars are gathered by women in areas close to the village, but plants used in communitarian celebrations, as well as the resin of *Bursera* spp., are carried out by men (Table 5). Journeys for gathering these products may take several hours or days and are considered dangerous activities, particularly those to obtain *Beaucarnea stricta*, *T*. *grandis*, and *Burmannia biflora* (Table 6). For the extraction of these plants, several techniques are common to prevent damage, such as leaving stems and main branches of the most valuable species (orchids, *B*. *biflora*, *B*. *stricta*, *C*. *alba*, and *L*. *glaucescens*). These techniques favor survival and resprouting of plants (Table 6). In total, 22 species that germinate and become spontaneously established in AFS are tolerated and their abundance enhanced, by leaving plants producing seeds or deliberately dispersing seeds in sites propitious for their growth (Tables 3 and 7). About 38 species receive special care such as irrigation, addition of organic matter, control of pests, and removal of competitors (Tables 3 and 7). Transplanting of juvenile plants of 26 species and propagation of 19 species is conducted with the purpose of having them closer to homes (mainly homegardens) in order to enjoy their beauty, having available their flowers, satisfying their curiosity to know how plants grow, and experimenting horticultural practices (Tables 3 and 6). We recorded experiments of in situ vegetative propagation of *B*. *biflora* and transplanting of several species of orchids and Bromeliaceae species (Tables 6 and 7). We in addition documented reasons why local people do not practice management. They consider unviable planting plants that are abundant or have special requirements and low probability of survival or those for whom they do not have information about plants’ requirements to survive and grow (Table 6) or when people have limitations of space for maintaining plants.

**Table 7:** Ecological and management parameters estimated for species considered in in-depth studies

	Management parameters							Management parameters					
ID	APe	VEA	LCi	Rep	HPa	Nea^a^	TAv^a^	CRe	MPr	Sel	MAFS	MLa^a^	MST^a^
Ceremonial													
Bbif	4	2	2	2	1, 1, 2, 9	1	2	1	2, 6	0	0.05	1	1, 3
Blon	2	2	2	2	5	3	2	0	1	1	0	0	1
Bstr	3.3	3	2	2	3	3.3	1	1, 2	2	1	0	0	1
Calb	1.5	3.5	2	2	1, 5	3.5	2	1	2	1	0	0	1
Dser	2	2	2	2	8, 9	4	1	1, 2	1, 2	1	0	0	1
Dspp	2.5	2	2	1	5, 9	3.5	2	1	2, 7	1	0.17	4	1, 3
Ekar	3.5	2	2	1	1, 8, 9	1	3	1	1, 2, 5, 6	1	0.63	1	1, 3
Erad	3.5	1	2	1	1, 8, 9	1.5	3	1	1, 2, 5, 6	1	0.32	1	1, 3
Lalb	5	2	2	1	1, 8, 9	1	3	1	1, 2, 5, 7	1	1.29	1	1, 3
Ldas	1.5	3	2	2	5	3	3	0	1	1	0	0	1
Lgla	2	2	2	2	5	3.5	1	1, 2, 3	2	0	0	0	1
Lmes	2	3	2	2	5	4	3	0	1	1	0	0	1
Mdep	1	1	2	1	10	1	1	0	1	0	0	0	1
Octa	3	4	2	2	5	3	1	1	2	1	0	0	1
Prub	4	0	2	2	5	1	2	0	1, 7	1	0.10	3	1, 3
Rmac	3.5	2	2	1	1, 8, 9	1	3	1	1, 2, 5, 6	1	0.30	1	1, 3
Spur	3.5	2	1	2	5	3	3	1	1, 2	1	0	0	1
Tgra	4	4	2	2	10	2	1	1	1	1	0	0	1
Tluc	2.7	2	2	2	9	4	2	0	1	0	0	0	1
Tusn	1	0	2	1	5, 10	4	1	0	1, 6	0	1.33	1	1, 3
Edible													
Acris	2	1.5	1	2	4, 10	4	2.5	0	1, 3	1	0.39		2
Ahyb	2	3	1	2	4, 10	4	2.5	1	1, 2, 3, 4, 5	1, 2	1.33		2
Aker	1	1.5	2	1	1, 1, 8	4	2	0	1, 3, 6	1	0.07		1, 2
Apot	2	2	2	2	1, 1, 3, 8, 10	2.8	1	1, 2	1, 3, 4, 5, 6, 7	1	1.16		1, 2, 3, 4
Bdul	1	2	2	1	1, 3, 8, 9, 9	4	2	1, 2	1, 2, 3, 5, 4, 6	1	2.01		1, 2, 3
Cber	3	3	1	2	4, 10	4.5	2.5	0	1, 3, 4	1	0.53		2
Crme	4	4	2	2	6	2	2	1	2	2	0.07		1, 2
Damb	2	4	1	2	3, 4	5	1.5	1	2, 3, 4, 5, 6, 7	1, 2, 3	0.62		2
Dser	4	2	2	2	8, 9	3	2	1, 2	1, 2	1	0		1
Lgla	2	2	2	2	5	3.5	1	1, 2, 3	2	0	0		1
Lspp	1	2	2	2	4, 6	3.5	2	0	1, 3, 6	0	0.84		1, 2, 3
Mspp	1.5	2	2	1	1, 6	4	3	0	1, 3, 6	0	0.68		1, 2, 3
Noff	3.5	1	2	2	4, 10	4	2	0	1	1	0		1
Olas	3	3	2	1	4, 6	5	2	0	1, 3, 4, 5, 6, 7	1, 2, 3	0.70		1, 2, 3
Ospp	2	2	2	1	3	4	2	0	1, 3, 6	1	1.59		1, 2, 3
Plin	1	2	1	2	10	4.3	2	0	1, 3, 4, 5, 6, 7	1	0.53		1, 2, 3
Pole	2.3	0.7	1	2	4, 10	3.6	2	0	1, 3, 4, 6	1	0.30		2
Pphi	2.5	4	1	2	6	4.5	2	0	2, 3, 4, 5, 6, 7	1, 2, 3	1.97		2
Pqua	3.5	1.5	2	1	9	1.5	1.5	0	1	1	0		1
Prud	2	2	2	2	4	4	2	1	2, 3, 4, 5, 7	1	0.24		1, 3
Medicinal													
Amai	3.5	1.5	2	2	5	3.5	2	1	1, 2, 3, 5, 6	1	0.37		1, 2, 3
Apsi	3	2	2	1	5	3	2	1	1, 2, 3	1	0.07		1, 2
Bsal	1	1	2	2	1, 1, 3, 5	5	1	0	1, 3, 5, 6	0	1.04		2
Clme	2.5	1.5	2	1	5	4	2	1	2, 5	1	0.60		1, 2
Cmex	4	2	2	2	5, 10	3	2	1	1, 2	1	0		1
Dcar	3	2	2	2	4	4	2	0	1, 3	1	0.07		1, 2
Gglu	2	1	2	2	5	4	2	0	1, 3	0	1.94		1, 2
Ginu	2	2	2	2	5, 10	4	2	1	2, 4, 5, 6	0	0.30		1, 2, 3
Loax	2.5	2.5	2	2	4, 5	3	2	1	1, 2	1	0		1
Mpar	1	2	1	2	10	5	2	0	1, 3, 4	1	0.72		2
Mpur	2	2	2	2	6, 9	4	2	0	1, 7	1	0.19		1, 2, 3
Mvul	1	2	2	2	4	5	1	0	1, 3	1	0.77		2
Ppen	1	1	2	2	10	5	2	0	3, 5	0	0.25		1, 2
Pros	1	1	2	2	3	4	2	0	1	1	0.67		1, 2
Rcom	1	1	2	2	0, 3	5	1	1	1, 2, 3, 4, 5, 6	1, 2	0.43		2
Spra	4	0	2	2	2	3	1	0	1, 3, 5, 6	0	0.10		1, 3
Tdif	2	0	2	2	5	2	2	0	1, 2	0	0		1
Tluc	2.7	2	2	2	9	4	2	0	1, 2	0	0		1
Apot	2	2	2	2	1, 1, 3, 8, 10	2.8	1	1, 2	1, 3, 4, 5, 6, 7	1	1.16		1, 2, 3, 4
Qacu	2	0	2	2	1, 5, 6, 7, 9	4	2	1, 2, 3	2, 5, 6, 7	1	0.03		1, 3

*ID* identification tag assigned to the species analyzed (check Table 3 to identify the species), *APe* abundance perception, *VEA* vulnerability to environmental factors, *LCi* life cycle, *Rep* reproduction, *HPa* harvested part, *Nea* nearness to harvest site, *TAv* temporal availability, *CRe* collective regulations, *MPr* management practices type, *Sel* artificial selection, *MAFS* management in AFS, *PrN* practice number, *MLa* maintaining labors, *MST* management system type

^a^Excluded variables and species in the performance of principal component analyses (PCA) and canonical correspondence analyses

Selective harvesting of plants based on use quality of their parts and absence of signs of herbivory are criteria for gathering most species documented. Although local people recognize at least five species with intraspecific varieties (identified according to flower color and forms), their use and management are indistinct (Tables 5 and 7). Except for *Tagetes erecta*, in which people select seeds for cultivation, and *Cosmos bipinnatus*, a species commonly producing violaceus ligula, people select the scarcer variety with white ligula.

Local regulations forbid extraction of plants for commercialization out of the village and establish restrictions in using some plants in communitarian celebrations (Table 7).

### Edible plants

We recorded 138 plant species used as food, 80 of them being wild and naturalized species and 20 considered as “basic” (Tables 3 and 4). The most valuable species are *Amaranthus hybridus*, *Porophyllum* spp., *Opuntia lasiacantha*, *Dysphania ambrosioides*, *Dasylirion serratifolium*, *Peperomia quadrifolia*, and *Physalis philadelphica*, which are consumed by more than 90% of households from 1 to 10 times per season (Fig. 2, Table 5). About 40 species are occasionally consumed where they are gathered and rarely carried to homes. These are the cases of *Chrysactinia mexicana* and *Cyrtocarpa procera*. Other 30 species are consumed occasionally, but it is considered that most of them were highly consumed in the past; these are the cases of *Chenopodium* spp., *Anoda cristata*, *Nasturtium officinale*, *Agave kerchovei*, and *A*. *potatorum*. Consumption of these plants has decreased due to higher presence of cultivated and processed food (Table 6). Other species are consumed occasionally by few households, as is the case of *L*. *glaucescens* which is used as a condiment or *Tagetes lucida*, *Lippia* sp., and *Turnera diffusa*, which in the past were commonly used as infusions and now were substituted by coffee.

Commercialization of managed weedy plants is allowed, and the most common is the green tomato *P*. *philadelphica*. Others occasionally commercialized are *O*. *lasiacantha* and cooked *A*. *potatorum* (Table 5). *P*. *philadelphica*, *C*. *mexicana*, *Porophyllum* spp., and *D*. *ambrosioides* are interchanged in local stores (Table 5). Local people share with relatives and friends part of the plants gathered or harvested (Tables 5 and 6). These are the cases of *D*. *serratifolium*, *P*. *quadrifolia*, *C*. *mexicana*, *P*. *philadelphica*, *D*. *ambrosioides*, *Opuntia* spp., *Porophyllum* spp., *A*. *potatorum*, and *A*. *hybridus*.

Most edible plant species are considered abundant (Table 7), but such abundance is associated with care during extraction or the management in crop fields and homegardens as it was documented for *P*. *philadelphica* (Table 6). Most species are considered vulnerable to environmental factors and pests (Table 7), and some of the most appreciated resources are perceived to be scarce. This is the case of *D*. *serratifolium*, which due to the scarcity of its inflorescences people stopped consuming them (Table 6).

Gathering of edible plants is generally carried out while practicing other activities—by men when plants are gathered from the forest and by women and children when plants are harvested from agricultural fields and homegardens. Gathering is the only practice for 30 species, which are immediately consumed (Table 3). Practices of care during gathering of useful parts aim to procuring plant survival, and these are carried out only in gathered plants and those under management (Tables 6 and 7). In order to ensure further availability, the abundance of seven species is enhanced by letting individual plants produce seeds and deliberately dispersing their seeds in appropriate places (Tables 3 and 7). At least 40 species are let standing in AFS, with the purpose of ensuring their availability (Tables 3, 6, and 7). For ensuring productivity and quality of products for consumption, 28 species receive irrigation, addition of organic matter, and exclusion from domestic animals (Tables 3 and 7). Nearly 20 species of weedy plants (among them *P*. *philadelphica* and *D*. *ambrosioides*) are transplanted into homegardens where people consider the plants to have better conditions for growing (Table 3). Other species occurring in the wild are transplanted to homegardens with the purpose of having them closer and to consume them for longer time (*Porophyllum* spp.) or for ornamental purposes (M*ammillaria* spp. and *Lantana* spp.) (Fig. 2, Tables 3 and 7). We recorded the deliberate propagation of 11 species through seeds and vegetative parts (Tables 3 and 6), as food (*Porophyllum* spp., *D*. *ambrosioides*, *P*. *philadelphica*, *Opuntia* spp.), for ornamental purposes, and for satisfying curiosity (*Oxalis* aff. *nelsoni*). Other species have started to be propagated, as is the case of *A*. *potatorum*, and others have had failed attempts (*L*. *glaucescens*, *C*. *mexicana*).

From seven species whose varieties are distinguished by morphology, flavor, and odor, we identified human selection in five of them; the preferred variants are tolerated, protected, or enhanced. For *D*. *ambrosioides*, *O*. *lasiacantha*, and *P*. *philadelphica*, we documented human selection favoring plants providing seeds or cladodes for cultivation (Tables 6 and 7).

Local customs and regulations forbid gathering wild edible plants for commercializing them out of the village, with the only exception of *Brahea dulcis* and *A*. *potatorum*, which are edible, but parts commercialized are destined for other uses. In the Communitarian Assemblies, we recorded discussions among local people and the Biosphere Reserve authorities for regulating and planning the use of *A*. *potatorum*, *B*. *dulcis*, and *D*. *serratifolium*. In the case of *L*. *glaucescens*, the Assembly decided to allow external people to extract it, but the permit stopped.

### Medicinal plants

We recorded 219 medicinal plant species, 178 of them being native and naturalized, and 22 considered “basic plants” (Tables 3 and 4). Currently, 85% of households use medicinal plants, generally complementing their healing treatment with massages, cupping therapy, and treatments by the national system of health through the local health center and private physicians. Women heads of families mainly make the decision on the appropriate treatment, while for traditional treatments, it is common to consult the relatives with more experience or one of the four traditional physicians in the village. The native plant species are mainly used for attending accidents (hurts, cuttings, twists, fractures, bites of poisonous animals), respiratory and stomach infections, pains, child tantrums, angers, “susto” (frightens), illnesses caused by “aires,” monitoring of pregnancy, and recovering of childbirth. Medicinal plants may be ingested and placed in affected body zones, steam baths, and “limpias” (ceremonies for cleaning the body and spirit).

Almost all medicinal plants are collected when they are needed, but for some of them (*Lippia oaxacana*, *T*. *lucida*, *T*. *diffusa*, *Chrysactinia mexicana*, *Ambrosia psilostachya*), people used to store dry materials or ask somebody else to get the needed plant (Fig. 2, Table 5).

No commercialization of medicinal plants was recorded; most medicinal plants are shared. Some plants are interchanged for plants with other uses, for instance, *Quercus acutifolia*, used and commercialized as fuelwood, and *A*. *potatorum* used in mescal production (Table 5). Except *C*. *mexicana* and *Pittocaulon praecox*, all medicinal plants are considered abundant, but dryness and frosts are factors affecting their availability (Table 7).

Gathering of wild medicinal plants is conducted by men and women; men gather plants occurring far away and women those occurring in homegardens. Gathering is the most common practice for all medicinal plants, and the only practice for 81 species (Tables 3 and 4). Practices for preventing damage of gathered plants are common on the most valuable plants (*Ambrosia psilostachya*, *Clinopodium mexicanum*, *C*. *mexicana*, *L*. *oaxacana*, *T*. *diffusa*, *T*. *lucida*, *Ageratina mairetiana*, *Grindelia inuloides*) (Table 7, Fig. 2). In AFS, 79 medicinal plants are let standing during vegetation clearing, as well as the 65 species distributed in homegardens (Table 3). Among them, *Ricinus communis*, *Marrubium vulgare*, and *Malva parviflora* are submitted to practices for controlling their abundance through weeding, similarly to 37 other species (Table 1). We recorded 31 species receiving care such as removal of competitors, addition of organic matter, and irrigation (Table 3). Abundance of nine species is enhanced by leaving plants to produce seeds or by spreading the seeds in appropriate sites for their germination and growth (Tables 6 and 7). We also documented the transplanting of 25 species, 8 of them from forests to homegardens (*G*. *inuloides*, *P*. *praecox*, and *A*. *mairetiana*) for their medicinal and ornamental uses (Tables 3 and 7). In addition, we recorded the propagation by seeds of 12 species, 2 of them mainly motivated to have them available when needed (*G*. *inuloides* and *Matelea purpusii*) (Fig. 2, Tables 3, 6, and 7). We documented failed attempts of transplanting and propagating six species, among them *A*. *mairetiana*, *A*. *psilostachya*, *G*. *inuloides*, and *L*. *oaxacana* (Table 6). Reasons for not transplanting individual plants from forests to homegardens were the following: lack of information about plant requirements and the supposition or experience that in changing habitat, plants do not survive and that using appropriate techniques of extraction or storing strategies are enough for ensuring their availability (Table 6). We recorded the recognition of varieties of three species, but people make differential use and management only of *R*. *communis* (Fig. 2, Table 7).

### Management intensity and risk

**Fig. 3:**
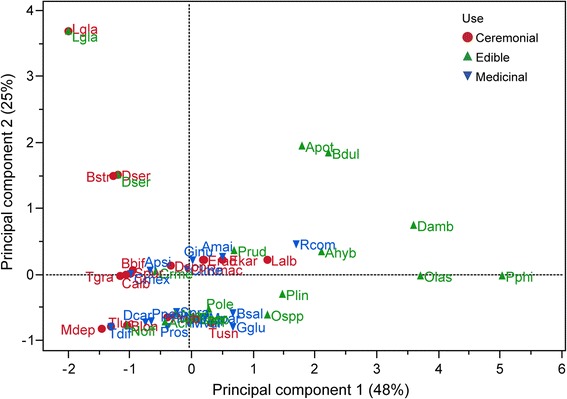
Management of ceremonial, edible, and medicinal plants according to principal component analysis (PCA). Edible plants tended to be managed most intensely, since ceremonial and medicinal plants are less intensely managed. Variation in spatial arrangement is mainly explained in the first principal component by management practices and collective regulations = (eigenvalues 0.631, −0.133 respectively) and by collective regulations and management in AFS in the second principal component (eigenvalues 0.986, −0.007 respectively)

Management intensity of edible, ceremonial, and medicinal plants studied is explained mainly by practices and communitarian regulations in the first component and by their presence in AFS in the second component (Fig. 3). Management intensity among use types was significantly different (KW *X* ^2^ = 9.9, df = 2, *p* = 0.007). Edible plants had the highest management intensity, most of them managed in AFS involving human selection, while most species used for ceremonial and medicinal purposes are gathered from forests and protected through communitarian regulations (Fig. 3).

**Fig. 4:**
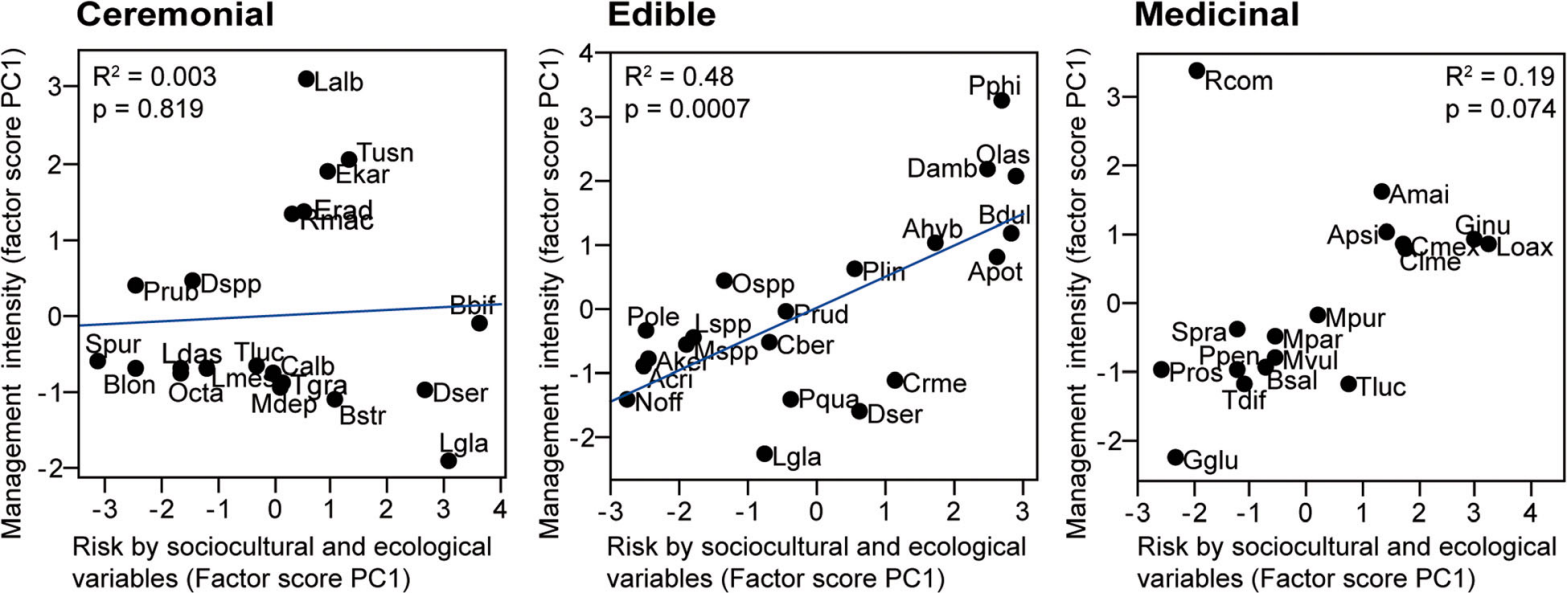
Relation between management intensity and risk. Regression analysis of the management intensity index as a function of the risk index due to sociocultural and ecological factors. Indexes were calculated as the scores of the first principal components performed by use type

**Fig. 5:**
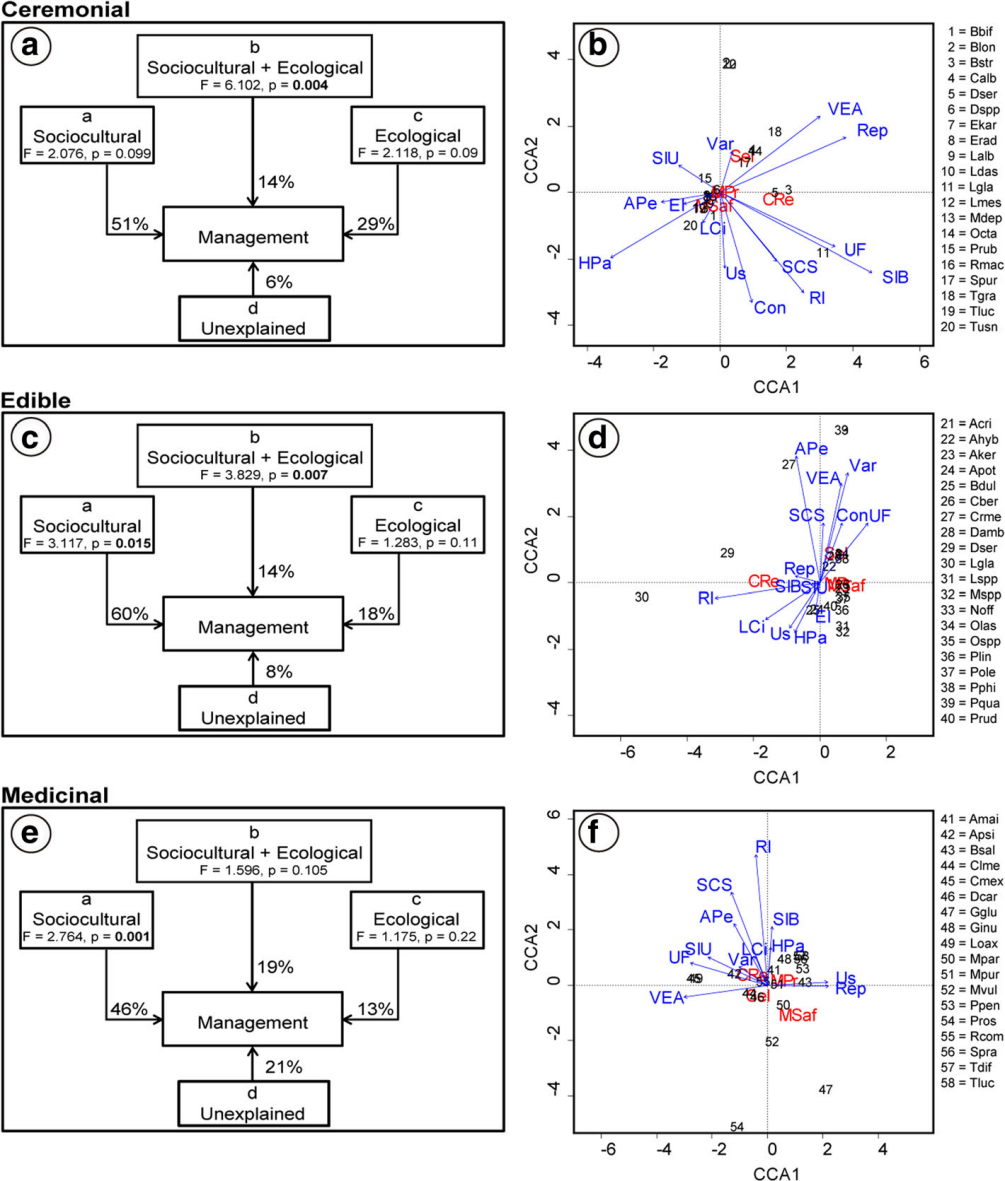
**a**–**f** Relative influence of risk due to sociocultural and ecological factors in plant management. Schemes show the relative influence of sociocultural and ecological factors and their interactions on management of ceremonial, edible, and medicinal plants based on partitioned canonical correspondence analyses (CCA) performed by type use. Ordination planes of CCA performed by type use show how species (numbers) and management variables (red words) are influenced by sociocultural and ecological variables (blue arrows)

In plants with ceremonial use, the regression analysis indicates no relation among management intensity and risk indexes (*R* ^2^ = 0.003, *p* = 0.819) (Fig. 4, Table 8). Partial CCA explains 95% of the variation of management, significantly explained by the intersection of sociocultural and ecological factors (14%) (Fig. 5a, Table 9). In plants with intermediate management intensity (Table 8), management regulated by collective rules occurs in plants basic for life and exclusively with sexual reproduction. These are the cases of *L*. *glaucescens*, *D*. *serratifolium*, *B*. *stricta*, and *T*. *grandis* (Fig. 5b, Table 9). Plants intensively managed (Table 8) in AFS are those providing several parts or the whole plant as resources, having asexual reproduction, and being abundant, like *Tillandsia usneoides*, or that are scarce, like *Laelia albida*, *Euchile karwinskii*, *Epidendrum radioferens*, and *Rhynchostele maculata* (Fig. 5b, Table 9).

**Table 8:** Management intensity and risk indexes of ceremonial, edible, and medicinal plants

Ceremonial			Edible			Medicinal		
ID	Management intensity index	Risk index	ID	Management intensity index	Risk index	ID	Management intensity index	Risk index
Bbif	0.12	0.62	Acri	0.13	0.27	Amai	0.29	0.47
Blon	0.05	0.28	Ahyb	0.47	0.54	Apsi	0.15	0.37
Bstr	0.19	0.47	Aker	0.14	0.27	Bsal	0.27	0.38
Calb	0.10	0.43	Apot	0.55	0.62	Clme	0.22	0.38
Dser	0.19	0.53	Bdul	0.61	0.57	Cmex	0.11	0.46
Dspp	0.19	0.32	Cber	0.18	0.41	Dcar	0.09	0.33
Ekar	0.29	0.42	Crme	0.15	0.57	Gglu	0.28	0.28
Erad	0.25	0.38	Damb	0.62	0.59	Ginu	0.24	0.43
Lalb	0.38	0.43	Dser	0.19	0.56	Loax	0.11	0.46
Ldas	0.05	0.39	Lgla	0.27	0.40	Mpar	0.21	0.33
Lgla	0.27	0.49	Lspp	0.20	0.35	Mpur	0.14	0.39
Lmes	0.05	0.35	Mspp	0.18	0.31	Mvul	0.17	0.33
Mdep	0.01	0.37	Noff	0.05	0.31	Ppen	0.11	0.31
Octa	0.10	0.36	Olas	0.58	0.61	Pros	0.13	0.25
Prub	0.13	0.34	Ospp	0.33	0.35	Rcom	0.41	0.41
Rmac	0.25	0.39	Plin	0.35	0.45	Spra	0.15	0.35
Spur	0.11	0.34	Pole	0.21	0.28	Tdif	0.03	0.33
Tgra	0.09	0.49	Pphi	0.75	0.62	Tluc	0.03	0.43
Tusn	0.23	0.36	Pqua	0.05	0.45			
Tluc	0.03	0.37	Prud	0.31	0.42			

Indexes were calculated based on the score of the first principal component of PCA performed by use type and variable kind, management variables for the management intensity index, and sociocultural and ecological variables for the risk index

*ID* identification tag assigned to the species analyzed; check Table 3 to identify the species

**Table 9:** Significance of explanatory variables on management associated with the canonical correspondence analyses (CCA) for ceremonial, edible, and medicinal plants

Risk variable	Ceremonial				Edible				Medicinal			
	Df	*X* ^2^	*F*	*p*	Df	*X* ^2^	*F*	*p*	Df	*X* ^2^	*F*	*p*
Sociocultural and ecological variables												
Uses number (Us)	1	0.017	3.24	0.103	1	0.014	1.98	0.105	1	0.016	1.46	0.175
SI basic plants (SIB)	1	0.190	35.41	*0.002*	1	0.008	1.09	0.219	1	0.021	1.84	0.11
SI by use type (SIU)	1	0.003	0.47	0.695	1	0.003	0.45	0.497	1	0.026	2.33	0.09
Consumption (Con)	1	0.029	5.35	*0.024*	1	0.013	1.80	0.125				
Use frequency (UF)	1	0.040	7.48	*0.012*	1	0.013	1.83	0.180	1	0.024	2.15	0.078
Economic interchange (EI)	1	0.010	1.85	0.22	1	0.006	0.79	0.389				
Reciprocity interchange (RI)	1	0.006	1.08	0.454	1	0.173	24.47	*0.002*	1	0.052	4.68	*0.007*
Recognized variants (Var)	1	0.008	1.41	0.345	1	0.048	6.79	*0.003*	1	0.014	1.24	0.269
Sociocultural strategies (SCS)	1	0.014	2.62	0.13	1	0.028	4.00	*0.034*	1	0.025	2.22	0.084
Abundance perception(Ape)	1	0.026	4.88	*0.045*	1	0.027	3.76	*0.035*	1	0.011	1.01	0.398
Harvested parts (HPa)	1	0.048	8.89	*0.008*	1	0.008	1.20	0.326	1	0.004	0.40	0.735
Life cycle (LCi)	1	0.002	0.37	0.734	1	0.005	0.74	0.465	1	0.001	0.06	0.981
Reproduction (Rep)	1	0.052	9.65	*0.002*	1	0.026	3.73	0.066	1	0.019	1.70	0.183
Vulnerability (VEA)	1	0.015	2.72	0.111	1	0.007	0.97	0.416	1	0.001	0.06	0.967
Residual	5	0.027			5	0.035			5	0.056		
Sociocultural variables												
Uses number (Us)	1	0.017	1.028	0.352	1	0.014	1.29	0.114	1	0.016	1.77	0.106
SI basic plants (SIB)	1	0.190	11.232	*0.005*	1	0.008	0.71	0.321	1	0.021	2.24	0.084
SI by use type (SIU)	1	0.003	0.148	0.925	1	0.003	0.29	0.680	1	0.026	2.83	0.051
Consumption (Con)	1	0.029	1.698	0.212	1	0.013	1.17	0.215				
Use frequency (UF)	1	0.040	2.374	0.131	1	0.013	1.19	0.217	1	0.024	2.61	*0.037*
Economic interchange (EI)	1	0.010	0.587	0.587	1	0.006	0.51	0.529				
Reciprocity interchange (RI)	1	0.006	0.343	0.808	1	0.173	15.89	*0.001*	1	0.052	5.68	*0.001*
Recognized variants (Var)	1	0.008	0.447	0.687	1	0.048	4.41	*0.029*	1	0.014	1.51	0.189
Sociocultural strategies (SCS)	1	0.014	0.832	0.487	1	0.028	2.60	0.099	1	0.025	2.70	*0.048*
Abundance perception(Ape)	10	0.170			10	0.109			10	0.092		
Ecological variables												
Abundance perception(Ape)	1	0.034	1.73	0.169	1	0.047	2.29	*0.047*	1	0.018	1.21	0.214
Harvested parts (HPa)	1	0.089	4.51	*0.023*	1	0.011	0.55	0.443	1	0.010	0.64	0.509
Life cycle (LCi)	1	0.001	0.05	0.983	1	0.024	1.18	0.196	1	0.002	0.14	0.953
Reproduction (Rep)	1	0.033	1.68	0.221	1	0.045	2.19	0.075	1	0.017	1.12	0.274
Vulnerability (VEA)	1	0.052	2.63	0.107	1	0.004	0.20	0.815	1	0.042	2.77	*0.03*
Residual	14	0.277			14	0.284			12	0.181		

Number of permutations = 999; *p* values in italics are significant at 0.05

In edible plants, the regression analysis indicates that there is a highly significant relation among management intensity and risk indexes (*R* ^2^ = 0.48, *p* = 0.0007) (Fig. 4, Table 8). Partial CCA explained 92% of the variation of management, significantly explained by sociocultural factors (60%) and the intersection of sociocultural and ecological factors (14%) (Fig. 5c, Table 9). Plants with the lowest management intensity (Table 8) are those protected through collective regulations, like *D*. *serratifolium* and *L*. *glaucescens*, which are shared among relatives and used in communitarian ceremonies, as well as in those gathered and perceived to be scarce, like *N*. *officinale*, *P*. *quadrifolia*, and *C*. *mexicana* (Fig. 5d, Table 9). Plants with the highest management intensity like *P*. *philadelphica*, *O*. *lasiacantha*, *A*. *hybridus*, and *D*. *ambrosioides* (Table 8) are those with different varieties, under human selection through several types of practices, considered to be abundant, shared among members of the community, and obtained through different strategies, among them interchange and commercialization (Fig. 5d, Table 9).

In medicinal plants, the regression analysis indicates no significant relation among management intensity and risk (*R* ^2^ = 0.19, *p* = 0.074) (Fig. 4, Table 8). Partial CCA explains 79% of the variation of management, mainly by sociocultural factors (46%) (Fig. 5e, Table 9). Plants with low risk like *Pinaropappus roseus* and *Gymnosperma glutinosum* are directly consumed by people who gather them and, along with *Marrubium vulgare*, occur in most of the homegardens and crop fields sampled. These plants are only gathered and let standing (Fig. 5e, Table 9). Management through collective regulations determining care during gathering was documented on *C*. *mexicana*, *L*. *oaxacana*, and *A*. *psilostachya*, with relatively high management intensity and risk (Table 8) associated to their value in reciprocity, use frequency, strategies for obtaining them, and the perception of vulnerability to environmental factors (Fig. 5e, Table 9).

## Discussion

### Management intensity

As we hypothesized, the gradient of management intensity is higher in edible plants, which are managed through different types of practices in AFS, more frequently, and involving human selection. Contrarily, plants used for ceremonies and as medicine are mostly tolerated or simply gathered. These general trends are similar to other reports for edible plants studied in the region which are managed with more complex practices than other useful plant species [6, 22, 43–45].

Collective regulations importantly influence the management intensity, but differently to that proposed for a general model of management intensity [46], the highest complexity of such regulations was observed in plants that are only gathered in areas of common access, such as the most valuable medicinal and ceremonial plants. For the contrary, edible species are mainly managed in AFS, where managers have higher control of access to plant resources. These differences reflect the trade-offs in managing natural resources of common use, as it has been discussed previously for edible plants of the region and for several resources of common use [13, 47]. In the case studied, this pattern is illustrated by the fact that collective regulations appear to be effective for plants culturally valuable but not for plant resources with high economic value. The inefficacy of collective regulations for plants like *A*. *potatorum* appears to be due to the lack of rules coherent with the weakening of local institutions for ordering the use of a resource of increasing demand [28]. The failure of regulations for achieving a balance between cost and benefit of its management has enhanced private management in sites for exclusive use. But also, external actors have promoted the reforestation in areas of common use [16, 26], actions that should be accompanied by strengthening the effectiveness of local institutions.

The selective management characterizes the high management intensity in plants under the three types of use, according to flavors, colors, and sizes of plants or plant parts, which indicates ongoing processes of domestication, which may have advanced expressions like in *P*. *philadelphica* or, rather incipient, like in *O*. *lasiacantha* and *R*. *communis* [5, 15, 48]. The indistinct use of species with varieties recognized such as *Chenopodium berlandieri* suggests that there exists a process of decreasing of consumption and interest in human selection, differently to what is happening with *A*. *potatorum*, whose propagation starts with gathering seeds from several sites where agaves are recognized to have differential productivity. Such contrasting situations indicate the dynamic aspect of the processes of domestication, in which changes in values, the introduction of new food or products, and changes in markets, among other factors, have direct effects on management of plant resources.

### Sociocultural and ecological factors and management intensity

As expected, management intensity in edible plants is associated with their high risk to disappear, compared to the pattern found in medicinal and ceremonial plants. However, in the analysis about how sociocultural and ecological factors influence on variation of management, we found a high variety of interactions. The economic value, which has been considered one of the most important factors motivating plant management [12, 13], was not significant in any of the systems studied. This result can be due to the low proportion of plant species that are interchanged through barter and commercialization, as well as the isolation of the community, a factor recognized to be significant for introducing non-timber forest products in markets [49]. Nevertheless, among the more intensely managed species, we recorded some whose management represents expenses (*P*. *philadelphica*) or their commercialization represents main incomes for households (*B*. *dulcis* and *A*. *potatorum*), which indicates a relation between management intensity and the economic role of plants in subsistence [46].

Consumption was only significant in ceremonial plants, explaining the gradient of management intensity according to the feasibility of propagation, which may be difficult in plants highly used (*Tillandsia grandis* and *Chiococca alba*), compared with species lowly used but having vegetative propagation that makes easy their management (*Plumeria rubra*, *Dahlia* sp.).

The perception of abundance and its interaction with cultural value and management feasibility was a meaningful factor for explaining gradients of management intensity of ceremonial and edible plants. For instance, *Peperomia quadrifolia*, a highly valued species as food, is only gathered following the principle of leaving part of the plant in order that it continues propagating, since it is scarce, but it has very specific habitat requirements. *Tillandsia usneoides* is intensely managed in homegardens, although it is abundant in forests, since it is easily propagated; *P*. *philadelphica*, a basic species, is considered abundant because of the effect of intense management. The examples suggest that the balance between the invested effort in management and benefits obtained according to needs is an important factor for making decisions [50].

The interchange of plant species related to reciprocity was significant for explaining variation of management of edible and medicinal plants. In both use types, the interchanged plants are the most valuable species. In the case of edible plants, our analysis explained the variation in the extremes of the gradient of management intensity; plants of difficult access are managed by collective regulations, and those intensely managed are in AFS. Among the medicinal plants, our analysis identified those species managed following collective regulations and stored, but in the case of emergency, people practice interchange. Importance of this factor coincides with other reports analyzing management of AFS, where it has been found that the social relations of local people are a main factor influencing biodiversity in these systems since plant species are introduced to the systems and because numerous species are maintained to be shared [44, 51, 52]. The study of these relations is covering importance for understanding management of AFS. We suggest that these may be considered for understanding management of species, since these are expressions of affect, respect, and solidarity, through which people construct social nets of mutual support that are part of the cultural identity and strategies for facing risks in their subsistence [28, 53–55].

In edible and medicinal plants, the interest for obtaining resources through sociocultural strategies influences the management intensity. Strategies like mobility for increasing the harvesting area and gathering for storing, among other practices, may determine some degree of risk on plants, which are placed through collective regulations and management practices.

The cognitive prominence by use type may be an indicator of resource quality, but this was no significant factor in our analysis. The perception about the quality of resources arose as a factor related to the place where plants grow. This aspect enhances plant management in AFS [51, 53, 56], which was documented with *P*. *philadelphica* and *O*. *lasiacantha*. In wild plants, this perception influences the communitarian regulations, as was recorded for *Bursera biflora*, whose resin is naturally produced and is preferred over that produced after cutting the stem [57].

### Management motives

Interactions between cultural importance, perception of scarcity, and feasibility of management suggest that several factors contribute to motivate management techniques, which was confirmed through the in-depth interviews qualitatively analyzed (Table 6). The worries expressed by people about the future availability of plants with ceremonial, edible, and medicinal uses suggest that uncertainty is a main motive determining management. Such worries can be explained because of the fact that in the analysis of cognitive prominence of plants considered as basic, people mentioned plants with the three uses, which means that they are considered indispensable elements of subsistence. This fact coincides with the general hypothesis of control of uncertainty as a main motive of management for ensuring resource availability [12]. However, the differences documented in types of management strategies and their intensity among use types may be due to the differential operation of other motives, as we hypothesized in this study.

Making easier the access to plants was an important motivation for transplanting or cultivating wild and weedy plants for the three use types analyzed. For edible and medicinal plants managed in homegardens, the main management motive is to have them close to home [22, 45, 58, 59]. And this is why people transplant and propagate plants that are naturally abundant into other ecosystems (e.g., *Porophyllum* spp.), protect with different labors the maintenance of *D*. *ambrosioides*, or tolerate weedy and ruderal plants like *Malva parviflora* and *Barkleyanthus salicifolius*. In ceremonial plants, the need to have flowers easily accessible is also an important motive for transplanting and propagating plants (for instance orchids and *Dahlia* spp.), but this motive is associated with the purpose of embellishing an area (60% of the ceremonial plant species are considered ornamental), a quality highly valued by the Ixcatec [6, 23, 24, 26, 52, 53, 60].

The symbolic value associated with plants and animals has been proposed relevant for making management decisions [8, 61, 62]. It is particularly important in plants used for ceremonies, like *B*. *biflora* [21], *L*. *glaucescens*, *Euchile karwinskii*, and other orchids, and may influence the perception of importance of being careful during their gathering and as a motive for propagation.

Our study suggests that ethical principles are important for regulating use and management in order to prevent damage to plants (Table 6), recognizing them as living beings with “the right to exist.” This is expressed in numerous tolerated plants with low cultural and economic value or even those without use [26]. Such criteria interact with others particularly in weedy and ruderal plants, with edible and/or medicinal uses such as *A*. *hybridus*, *M*. *parvifolia*, *R*. *communis*, and *M*. *vulgare* in which the perception of their potential as invasive plants determines a balance of efforts for maintaining and removing them [23, 26]. Other motives identified in the maintenance of homegardens [59, 63, 64], such as experimental curiosity, were mentioned by people in order to develop continual innovation in management techniques.

This study aspires to contribute to understand the multifactorial influence of social and ecological aspects on decisions for managing plant resources [26, 65] with different purposes. It is clear from this and other studies that management of edible resources are mainly influenced by factors associated with availability of food or means for obtaining it, whereas medicinal plants, which are consumed less frequently, involve quality rather than quantity, and ritual plants involve symbolic aspects. The three groups of plants involve management, but the intensity required in each case varies. However, some plant resources are particularly valuable because of their multi-functionality [65]; these are species that in this study are called “basic” by local people and are outstandingly important resources receiving the greatest management intensity.

Ixcatlán is the only site in the world where the Ixcatec language is spoken, and only 15 persons speak this language. Our ethnobiological studies look for contributing to efforts of a linguistic group working in favor of conserving and recovering this language. Information recovered in this study includes audio and image systems that have helped to produce educative materials useful for teachers in schools for teaching the Ixcatec language. In addition, the information about resource use, and particularly about management techniques, are helpful for planning actions for ordination, conservation, and recovering forest areas and resources, as well as agroforestry systems, which are part of the biocultural heritage of the Ixcatec for the Ixcatec people, people of the Biosphere Reserve Tehuacán-Cuicatlán, and the Mexican people.

## Conclusions

For managing edible, medicinal, and ceremonial plants, the Ixcatec have developed a broad variety of practices and regulations. Management strategies are motivated as responses to uncertainty in their availability and other motivations like embellishing an area, satisfying customs, emotions, and curiosity operating simultaneously in the decisions. Such a variety of factors is associated to a well-being premise combining both material and spiritual needs, as well as maintaining social relations and traditions that are part of the Ixcatec cultural identity [27, 50].

The highest management intensity in economic valuable species, mainly edible plants, indicates that uncertainty is significant in indispensable plants for satisfying subsistence needs. However, species of medicinal and ceremonial uses and some edible plants are managed through diverse management practices without response to abundance perception. These facts make necessary to analyze more deeply how needs, worries, external pressures, and management responses are articulated with subsistence strategies of households and communities in these processes, as well as the role of systems of ethical values and traditional regulation institutions.

Our study confirms the importance of sociocultural factors associated with use and interchange of resources, and ecological processes influencing the vulnerability and feasibility of managing them [12, 16, 17]. The multiple criteria may be useful to analyze conditions guiding early management motives that modeled the biocultural heritage of peoples of the Tehuacán Valley.

## Abbreviations

AFS: Agroforestry systems
CCA: Canonical correspondence analyses
PCA: Principal component analyses
TEK: Traditional ecological knowledge
UNAM: Universidad Nacional Autónoma de México
